# The handling of missing data in trial-based economic evaluations: should data be multiply imputed prior to longitudinal linear mixed-model analyses?

**DOI:** 10.1007/s10198-022-01525-y

**Published:** 2022-09-26

**Authors:** Ângela Jornada Ben, Johanna M. van Dongen, Mohamed El Alili, Martijn W. Heymans, Jos W. R. Twisk, Janet L. MacNeil-Vroomen, Maartje de Wit, Susan E. M. van Dijk, Teddy Oosterhuis, Judith E. Bosmans

**Affiliations:** 1 Department of Health Sciences, Faculty of Science, Vrije Universiteit Amsterdam, Amsterdam Public Health Research Institute, Van der Boechorststraat 7, 1081 BT Amsterdam, The Netherlands; 2 Department of Epidemiology and Data Science, Amsterdam UMC, Amsterdam Public Health Research Institute, Amsterdam, The Netherlands; 3 Section of Geriatrics, Department of Internal Medicine, Amsterdam Public Health Research Institute, Amsterdam UMC, University of Amsterdam, Amsterdam, The Netherlands; 4 Department of Medical Psychology, Amsterdam UMC, Vrije Universiteit, Amsterdam Public Health Research Institute, Amsterdam, The Netherlands; 5 Netherlands Society of Occupational Medicine (NVAB), Utrecht, The Netherlands

**Keywords:** Cost–benefit analysis, Longitudinal studies, Epidemiologic methods, Computer simulation, C15

## Abstract

**Introduction:**

For the analysis of clinical effects, multiple imputation (MI) of missing data were shown to be unnecessary when using longitudinal linear mixed-models (LLM). It remains unclear whether this also applies to trial-based economic evaluations. Therefore, this study aimed to assess whether MI is required prior to LLM when analyzing longitudinal cost and effect data.

**Methods:**

Two-thousand complete datasets were simulated containing five time points. Incomplete datasets were generated with 10, 25, and 50% missing data in follow-up costs and effects, assuming a Missing At Random (MAR) mechanism. Six different strategies were compared using empirical bias (EB), root-mean-squared error (RMSE), and coverage rate (CR). These strategies were: LLM alone (LLM) and MI with LLM (MI-LLM), and, as reference strategies, mean imputation with LLM (M-LLM), seemingly unrelated regression alone (SUR-CCA), MI with SUR (MI-SUR), and mean imputation with SUR (M-SUR).

**Results:**

For costs and effects, LLM, MI-LLM, and MI-SUR performed better than M-LLM, SUR-CCA, and M-SUR, with smaller EBs and RMSEs as well as CRs closers to nominal levels. However, even though LLM, MI-LLM and MI-SUR performed equally well for effects, MI-LLM and MI-SUR were found to perform better than LLM for costs at 10 and 25% missing data. At 50% missing data, all strategies resulted in relatively high EBs and RMSEs for costs.

**Conclusion:**

LLM should be combined with MI when analyzing trial-based economic evaluation data. MI-SUR is more efficient and can also be used, but then an average intervention effect over time cannot be estimated.

**Supplementary Information:**

The online version contains supplementary material available at 10.1007/s10198-022-01525-y.

## Introduction

Decisions about the implementation and/or reimbursement of new healthcare interventions increasingly rely on evidence of their cost-effectiveness compared to one or more alternative interventions, preferably usual care, to optimize the use of scarce healthcare resources [1, 2]. Economic evaluations seek to provide this information by relating the difference in costs between healthcare interventions to the difference in effects [2]. In many cases, economic evaluations are performed alongside clinical trials, which are then referred to as trial-based economic evaluations [3]. Important advantages of trial-based economic evaluations include the prospective collection of cost and effect data as well as the use of patient-level information to draw inferences about the cost-effectiveness of healthcare interventions [3].

Missing data are common in clinical trials as participants may skip questions, follow-up assessments, and/or drop out of the study [4]. In trial-based economic evaluations, costs are calculated as the sum of numerous cost components that are measured at different time points. Thus, if one cost component is missing, costs cannot be calculated [2, 3, 5]. In the literature, three different missing data mechanisms are distinguished that describe the association between missing data and observed and unobserved variables [6]. Missing Completely At Random (MCAR) assumes missing data to occur by chance. Hence, the missing data are not associated with observed or unobserved variables. Missing At Random (MAR) occurs when the missing data are associated with observed variables, but not with unobserved variables. Missing Not At Random (MNAR) occurs when missing data sre associated with unobserved variables [6].

Historically, missing data in trial-based economic evaluations were handled by simply deleting participants with missing values (i.e., complete-case analysis). However, deleting missing cases from the analysis potentially biases estimates, as systematic differences may exist between subjects with missing and complete data [5–7]. Other methods to account for missing data in trial-based economic evaluations include “naïve” imputation methods, such as mean imputation and last observation carried forward. The use of “naïve” imputation methods is discouraged, because these methods do not account for the uncertainty related to filling in the missing values like other more advanced methods do [7–11]. Advanced methods, such as Multiple Imputation (MI) and Longitudinal Linear Mixed-model analysis (LLM) with maximum likelihood estimation are therefore considered more valid and are increasingly used for handling missing data [7–10, 12, 13].

The advantage of MI is that it estimates the total variance of the summary statistic considering the within- and between-imputation variance, reflecting the uncertainty around estimated missing values [13]. Another key advantage of MI is the possibility to include auxiliary variables in the imputation model that may be not relevant for the analysis model. This can help in improving the precision of the estimates and adjust for important missingness predictors. A possible disadvantage of such an approach, however, is the change of mis-specifying the imputation model, which may in turn lead to incorrect results. Another possible disadvantage of MI is the added computational complexity, because outcomes need to be estimated for all imputed datasets an then they are pooled to obtain overall estimates. Also, MI may lead to unstable results when no sufficient imputations are performed [14].

Previous studies have shown that when opting for LLM, in case of reasonably normally distributed outcomes, MI is not necessary to obtain unbiased estimates, because the maximum likelihood function uses all observed data and produces unbiased estimates under the MAR assumption [14, 15]. Faria et al. [5] suggested that MI is not required prior to LLM in trial-based economic evaluations either, but this has never been empirically tested. This is important because there are three distinct statistical challenges to trial-based economic evaluations that may affect the performance of LLM to deal with missing data: 1) costs are typically heavily right-skewed; 2) costs are cumulative sums over time, and 3) costs and effects are correlated [15]. This study aimed to bridge this gap in knowledge by assessing whether MI of missing variables prior to LLM increases its performance when analyzing longitudinal cost and effect data. Additionally, the impact of using either one of these approaches on cost-effectiveness estimates was assessed using empirical data.

## Methods

To assess whether MI of missing values is required prior to LLM, a simulation study was conducted. SUR and mean imputation were added as analytic strategies to assess the relative performance of the main models (i.e., LLM and MI-LLM) versus (simpler) alternative approaches. Mean imputation was added because the results of Sullivan et al. [17] suggest that MI is not the only acceptable way to handle missing data in RCTs and that simpler approaches, such as mean imputation, may also have satisfactory performance. SUR was added, because with LLM the possible correlation between costs and effects is neglected, whereas SUR can account for this correlation through correlated error terms [16, 17]. In total, we compared six different methodological strategies; (i) LLM alone (LLM), (ii) mean imputation combined with LLM (M-LLM), (iii) MI combined with LLM (MI-LLM), (iv) seemingly unrelated regression alone (SUR-CCA), (v) mean imputation combined with SUR (M-SUR), and (vi) MI combined with SUR (MI-SUR). Additionally, two empirical datasets were used to evaluate the external validity of the results [18, 19].

### Simulated datasets

#### Complete data generation

Two thousand complete datasets were generated using the Simstudy R package (R statistical software–version 3.5.2) [20]. The parameters used for generating variables were based on previous trial-based economic evaluations [21, 22] and the empirical datasets [18, 19], and are summarized in Table 1. The R code for data generation can be found in Supplementary material 1.

**Table 1:** Parameter values

Parameters	Value	Motive
Age	Mean of 40 years with a 5-year difference between treatment groups (trt) and a variance of 140. Data were simulated using a normal distribution and a minimum of 18 and maximum of 99 years Formula: age = "40 + (5*trt)", variance = 140, dist = "normal" data$age < = 18, 18, data$age data$age > = 99, 99, data$age	Mean age and distribution were in line with those that might be observed in empirical trial-based economic evaluations. The variance was tweaked up until the point of getting a standard deviation similar to that encountered in empirical data[18, 19, 21, 22] . A slight baseline imbalance was simulated to enable the generation of a plausible MAR assumption
Gender	The mean proportion of male subjects was 52% and the proportion of female subjects 48%. Data were simulated using a binary distribution Formula: Gender = "0.52 + (0.4*trt)", dist = "binary"	The proportion of male subjects was in line with those that might be observed in empirical trial-based economic evaluations[18, 19, 21, 22]. A slight baseline imbalance was simulated to enable the generation of a plausible MAR assumption
Utility values at baseline (uT0)	uT0 was generated dependent on age and gender, with a variance of 0.002, and a gamma distribution with logit link function Formula: uT0 = "0.2 + (0.0045 * age) + (0.025 * gender)", variance = 0.002, dist = "gamma", link = "logit"	The dependency on age and gender allowed for the generation of a plausible MAR assumption. A gamma distribution with a logit link function and a variance of 0.002 were used as these parameters allowed for the generation of utility values ranging from 0 to 1. In doing so, we made sure that the average values and variances were in line with those that might be encountered in empirical trial-based economic evaluations[18, 19, 21, 22]
Costs at baseline (cT0)	Mean cT0 was generated dependent on age and gender, with a variance of 0.15, and a gamma distribution with logit link function Formula: "50 + (15*age) + (100*gender)", variance = 0.15, dist = "gamma", link = "logit" cT0 and uT0 were generated with a negative correlation of -0.5 data <−addCorFlex(data, defb, rho = − 0.5)	The dependency on age and gender allowed for the generation of a plausible MAR assumption. A gamma distribution with a logit link function and a variance of 0.15 were used to generate baseline costs similar to those that might be encountered in empirical trial-based economic evaluations[18, 19, 21, 22] A negative correlation between costs and utilities was set, as higher costs are generally associated with lower health-related quality of life[18, 19, 21, 22]
Utility values at T1 (uT1) and follow-up utilities	Mean uT1 was generated dependent on uT0, age, and gender with a difference of 0.04 between treatment groups and a variance of 0.002 following a gamma distribution with logit link function Formula: uT1 = "uT0 + (0.04* trt) + (0.002 * age) + (0.01 * gender)", variance = 0.002, dist = "gamma", link = "logit" Utilities at time points T2, T3, and T4 were generated based on uT1 with a correlation of 0.9 and a variance of 0.005 Q <—matrix(c(1.0,0.9,0.9,0.9,1.0,0.9,0.9,0.9,1.0), nrow = 3) data <—addCorGen(dtOld = data, idvar = "id", nvars = 3, corMatrix = Q, dist = "normal", param1 = "uT1", param2 = "variance", cnames = "uT2, uT3, uT4")	A difference in follow-up utility values of 0.04 was generated per time point based on the minimally important difference for this outcome that has previously been found to range from 0.03 to 0.52[54, 55] A gamma distribution with a logit link function and a variance of 0.002 were used to generate follow-up utilities similar to those that might be encountered in empirical trial-based economic evaluations[18, 19, 21, 22]
Costs at T1 (cT1) and follow-up costs	Mean cT1 was generated dependent on cT0, age, and gender with a difference of €62.5 between treatment groups per time point and a variance of 0.15 following a gamma distribution with logit link function Formula: "cT0 + (62.5*trt) + (1*age) + (10*gender)", variance = 0.15, dist = "gamma", link = "logit" Costs at time points T2, T3, and T4 were generated based on cT1 with a correlation of 0.7 and a variance of 0.1	A difference in follow-up costs of €62.5 was simulated per time point to generate a total cost difference during follow-up of €250 A gamma distribution with a logit link function and a variance of 0.15 were used to generate follow-up costs similar to those that might be encountered in empirical trial-based economic evaluations[18, 19, 21, 22]

A total of 600 subjects per dataset was generated. An intervention to control ratio of 52:48 was simulated to resemble slightly unbalanced empirical datasets using a binomial distribution. A complete set of baseline variables was generated, including age, gender, costs (cT0), and utility values (uT0; i.e. a measure of health-related quality of life, HRQoL) [23]. Age was generated using a normal distribution, gender using a binomial distribution, while costs and utility values were generated with a gamma distribution (Table 1) [24]. At baseline, age and gender were positively related with costs and utility values, indicating that we assumed older subjects and men to have higher costs and utility values than younger subjects and women, respectively. To create a plausible MAR assumption, age and gender were slightly imbalanced, but we made sure that these imbalances were small and in line with those encountered in previous trial-based economic evaluations [19, 21, 22, 25]. Additionally, we assumed the correlation between costs and utility values to be about − 0.50 [26, 27]. Such a negative correlation might appear when subjects with a lower health-related quality of life (i.e. lower utility values) have higher treatment costs, because they were less healthy to begin with [27].

Follow-up cost and utility values were simulated, with a true total cost and quality-adjusted life-year (QALY) difference during the complete-duration of follow-up of 250 euros and 0.04 QALY, respectively (Table 1, Supplementary material 1). This was done by first generating costs and utility values at 3-month follow-up (i.e., cT1 and uT1), based on the subjects’ baseline cost and utility values (i.e., cT0 and uT0) as well as their age, gender, and treatment allocation (trt). Then, all other follow-up cost and utility values (i.e., 6, 9, and 12-month follow-up) were extrapolated from the subjects’ 3-month follow-up values, using a correlation between time points of 0.7 for costs and 0.9 for utility values and a compound symmetry correlation structure (i.e., correlations between subsequent time-points were assumed to be the same).

#### Missing data generation

We assumed baseline data to be completely observed for all subjects, which is often the case in trial-based economic evaluations [19, 21, 22, 25]. Missing follow-up cost and utility values were generated under the assumption that the missingness of data was solely due to drop-out, resulting in monotone missing data patterns only (Supplementary material 2). This means that subjects were assumed to either complete the study, and hence all follow-up assessments, or to drop-out after 3, 6, 9, or 12-months and have missing data from that time point on. We acknowledge that intermittent missingness patterns (e.g., non-monotone) may also exist in empirical data. However, for the sake of simplicity, and considering previous literature [28, 29], we opted to simulate monotone missing data patterns only.

To generate follow-up missing values, a missing data indicator ${m}_{ij}$ was generated for every subject *i* (*i* = 1, …, *N* = 600) at time point *j* (*j* = 1, …, 4) using a binomial distribution and a logit link function to model the linear dependence between the probability of missing data and its predictive variables (i.e., age, gender, cT0, uT0, trt).

$${m}_{ij} \sim Binomial\left({\pi }_{mij}\right),$$

1 $$logit\left({\pi }_{mij}\right)= {\beta }_{0}+ {\beta }_{1}{age}_{i}+ {\beta }_{2}g{ender}_{i}+ {\beta }_{3}{uT0}_{i}+ {\beta }_{4}{cT0}_{i}+ {\beta }_{5}{trt}_{i}+ {\varepsilon }_{i},$$

where ${\pi }_{mij}$ is the probability of a subject *i* having missing data at time point *j*. The intercept (${\beta }_{0})$ and coefficients (${\beta }_{1, }{\beta }_{2, }{\beta }_{3, }{\beta }_{4, } {\beta }_{5})$ of covariates were tweaked to generate a plausible MAR assumption and datasets with 10, 25, and 50% missings. ${\varepsilon }_{i}$ is the error term (Supplementary material 2) [28]. Then, at the respective time point *j*, all complete follow-up cost and utility values were replaced by missing values according to ${m}_{ij}$. This process was repeated for all consecutive time points amongst the subset of individuals still in the study [28]. Thus, the “missingness” of costs and utility values at all time points was conditional on age, gender, as well as their baseline values. Subjects in the intervention group were assumed to be 4, 2, and 1 times more likely to have missing values compared to their control group counterparts for the datasets with 10, 25, and 50% missing data, respectively. This was done to strengthen the MAR assumption. The MAR assumption in our simulation differs from the MNAR assumption, because the ${\pi }_{mj}$ was independent of the partially observed follow-up cost and utility values [6, 7].

#### Number of simulations

The number of required simulated data sets (*n*_*sim*_) was calculated using the Monte Carlo standard error of the expected coverage rate [30]. The Monte Carlo standard error quantifies simulation uncertainty and provides an estimate of the standard error for simulation performance measures when using a finite number of simulations [30]. The coverage rate of the confidence intervals, that uses information of the Monte Carlo standard error represents the probability that a confidence interval contains the ‘true’ value. To estimate the coverage rate with an acceptable degree of imprecision, a total of 1900 simulated datasets (rounded up to 2000) was found to be needed based on a maximal Monte Carlo standard error of 0.5 and an expected coverage rate of 95%.

### Data analyses

Data analyses were performed in StataSE 16® (StataCorp LP, CollegeStation, TX, US). All Stata codes can be found in Supplementary material 2, 3, and 4.

#### Methodological strategies

Longitudinal Linear Mixed-model analysis (LLM)–Two separate LLMs were performed, including one for costs and one for utility values:

$${Costs}_{ij}= {\beta }_{1c}{time}_{j}+{{\beta }_{2c}{trt}_{i}+\beta }_{3c}{time}_{j}{trt}_{i}+ {\beta }_{4c}{cT0}_{i}+{\beta }_{5c}{{time}_{j}cT0}_{i}+ {{\beta }_{6c}{age}_{i}+\beta }_{7c}{time}_{j}{age}_{i}+{ {\beta }_{8c}{gender}_{i}+\beta }_{9c}{{time}_{j}gender}_{i}+ {\omega }_{ci}+ {\varepsilon }_{cij}$$

$${Utility}_{ij}= {\beta }_{1u}{time}_{j}+{{\beta }_{2u}{trt}_{i}+\beta }_{3u}{time}_{j}{trt}_{i}+ {\beta }_{4u}{uT0}_{i}+{\beta }_{5u}{{time}_{j}uT0}_{i}+ {{\beta }_{6u}{age}_{i}+\beta }_{7u}{time}_{j}{age}_{i}+{{\beta }_{8u}{gender}_{i}+\beta }_{9u}{{time}_{j}gender}_{i}+ {\omega }_{ui}+ {\varepsilon }_{uij}$$

2 $$\omega_{i} \sim Normal(0,\sigma_{\omega }^{2} ),\;\varepsilon_{ij} \sim Normal(0,\sigma_{\varepsilon }^{2} )$$

where ${Costs}_{ij}$ and ${Utility}_{ij}$ represent the cost and utility values of subject *i* (*i* = 1, …, *N* = 600) at time point *j* (*j* = 1, …, 4). The model parameters include the coefficients ${{\beta }_{1, \dots , }\beta }_{9}$ of covariates, including various – by ${time}_{j}$ interactions. Moreover, ${\omega }_{ci}$ and ${\omega }_{ui}$ represent the random intercepts and ${\varepsilon }_{cij}$ and ${\varepsilon }_{cij}$ the error terms at each time point *j* for costs and utility, respectively. Both ${\omega }_{i}$ and ${\varepsilon }_{ij}$ follow a normal distribution [28, 31, 32].

To calculate the total cost difference between treatment groups, information was extracted on the average cost differences per time point, after which all follow-up cost differences were summed. To estimate the difference in QALY between treatment groups, information was extracted on the average utility differences per time point, after which the area under the curve method was applied[23]. Detailed information on model specification can be found in Supplementary material 3.

Mean Imputation combined with LLM (M-LLM)–In this strategy, missing cost and utility values were replaced by the mean values from the available cases at each time point (i.e., unconditional mean imputation) [5]. Subsequently, two separate LLMs were fitted as outlined under LLM (Supplementary material 3).

Multiple imputation combined with LLM (MI-LLM): in this strategy, missing cost and utility values were first imputed using Multivariate Imputation by Chained Equations (MICE; FCS-standard) [33] with Predictive Mean Matching (PMM) [34]. With PMM, a case with one or more missing values is matched with a number of cases with complete data (i.e., donor observations) [34]. The characteristics used to match an observation with missing values with donor observations are the variables specified in the imputation model, which in our case included age, gender, cT0, and uT0. Subsequently, a value is randomly drawn from the donor observations that have an observed value for that variable [34]. The MICE algorithm then uses this random value to fill in missing data in an iterative process until the pre-specified imputation model converges [33]. A set of 5 donors with complete data was used for the matching (i.e., a k-nearest neighbor [knn] of 5) [34]. The imputation model was stratified by treatment group (i.e., trt) [17]. In total, 10 datasets were imputed for datasets with 10% and 25% missing data, and 20 for datasets with 50% missing data. This was done to ensure that the loss-of-efficiency was smaller than 0.05 [33]. Subsequently, two separate LLMs were fitted per imputed dataset as outlined under LLM, after which pooled estimates were obtained using Rubin’s rules [6] (Supplementary material 3).

Seemingly Unrelated Regressions—Complete Case Analysis (SUR-CCA) – In this strategy, total costs and QALY were calculated by adding costs at each time point and using the area under the curve method, respectively [23]. Subsequently, a seemingly unrelated regressions (SUR) model was fitted. A SUR model consists of two separate regression equations, e.g. one for total costs and one for QALY, while simultaneously correcting for their possible correlation through correlated error terms [16, 35]. With the current strategy, only subjects with completely observed data were analyzed (i.e., a complete-case analysis).

$${Costs}_{i}= {\beta }_{0c}+ {\beta }_{1c}{trt}_{i}+ {\beta }_{2c}{cT0}_{i}+ {\beta }_{3c}{age}_{i}+ {\beta }_{4c}{gender}_{i}+{\varepsilon }_{ci}$$

$${QALY}_{i}= {\beta }_{0q}+ {\beta }_{1q}{trt}_{i}+ {\beta }_{2q}{uT0}_{i}+ {\beta }_{3q}{age}_{i}+ {\beta }_{4q}{gender}_{i}+ {\varepsilon }_{qi}$$

3 $$\left(\begin{array}{c}{\varepsilon }_{ci} \\ {\varepsilon }_{qi}\end{array}\right)\sim Normal\left(\left(\begin{array}{c}0\\ 0\end{array}\right), \left(\begin{array}{cc}{\sigma }_{c}^{2}& {\sigma }_{cq}\\ {\sigma }_{cq}& {\sigma }_{q}^{2}\end{array}\right)\right)$$

where ${Costs}_{i}$ and ${QALY}_{i}$ are the observed total costs and QALY during follow-up of subject *i* (*i* = 1, …, N). ${\beta }_{0c}$ and ${\beta }_{0e}$ represent the models’ intercept,${\beta }_{1c}$ and ${\beta }_{1q}$ represent the regression coefficients of the independent variable ‘treatment group’ (trt). ${\beta }_{2c}\dots {\beta }_{4c}$ and ${\beta }_{2q}\dots {\beta }_{4q}$ represent the regression coefficients for baseline values, age, and gender, ${\varepsilon }_{ci}$ and ${\varepsilon }_{ei}$ represent the correlated error terms for costs and QALY, respectively [16, 35].

Mean Imputation combined with SUR (M-SUR): in this strategy, follow-up missing cost and utility data were replaced by the mean values from the available cases at each time point (i.e., unconditional mean imputation) [5]. Subsequently, total costs and QALYs were calculated, and SUR analyses were performed as outlined under SUR (Supplementary material 3).

Multiple imputation combined with SUR (MI-SUR) – In this strategy, follow-up missing cost and utility data were first imputed using multivariate imputation by chained equations (MICE) as outlined under MI-LLM [33]. Then, total costs and QALYs were calculated and a SUR was fitted per imputed dataset as outlined under SUR, after which pooled estimates were obtained using Rubin’s rules (Supplementary material 3) [33].

#### Comparison of the methodological strategies

The performance of the methodological strategies was assessed with regard to costs and QALY differences using the following performance measures: empirical bias (EB), root-mean-square error (RMSE) and coverage rate (CR) [30]. Performance measures were calculated by comparing the ‘true’ values (i.e., estimand, [$\theta$]) with the estimated values $\left(\widehat{\theta }\right)$ obtained from the methodological strategies conducted over the 2000 simulated datasets. Monte Carlo standard errors were estimated for each performance measure to quantify the uncertainty of these measures due to using 2000 simulated datasets [30] (Supplementary material 3).

(i) Empirical bias (EB) represents the average difference between $\widehat{\theta }$ and $\theta$. This performance measure estimates whether a method targets θ on average and must, therefore, be small.

4 $$EB=\frac{1}{{n}_{sims}} \sum_{i=1}^{{n}_{sims}}\left(\widehat{{\theta }_{i}}-\theta \right)$$

(ii) Root-mean-square error (RMSE) represents the square root of the difference between $\widehat{\theta }$ and $\theta$.

5 $$RMSE=\sqrt{\frac{1}{{n}_{sims}} \sum_{i=1}^{{n}_{sims}}{\left(\widehat{{\theta }_{i}}-\theta \right)}^{2}}$$

The mean-square error (MSE) is a measure of accuracy that combines the bias and variance in a single measure [36]. For easier interpretation, we report the square root of the MSE, to express it on the same scale as costs and QALYs [36].

(iii) Coverage rate (CR) represents the percentage of times that the $\theta$ is covered by the estimated 95% CI of $\widehat{\theta }$.

6 $$CR=\frac{1}{{n}_{sims}} \sum_{i=1}^{{n}_{sims}}1\left(\widehat{{CB}_{lower, i}}\le \theta \le \widehat{{CB}_{upper, i}}\right)$$

The Monte Carlo standard error distance from the nominal value of 0.95 was used as a criterion of poor coverage [37]. It is worth to note that CR alone may mislead conclusions about the accuracy of methodological strategies, because high variances in estimates can lead to high CR [30, 38]. Therefore, in this study, CR was evaluated jointly with the other two performance measures [30].

In addition to assessing the performance of the methodological strategies for handling missing data (see Sect. 2.2.1), we assessed the performance of LLM and SUR in the complete datasets. This was done to have a better understanding of their performance in the context of a trial-based economic evaluation in general. That is, before examining their performance for handling missing data).

### Empirical datasets

#### Description of the datasets

Data from two pragmatic randomized controlled trials were used in addition to the simulated data. In the first trial (empirical dataset 1), the cost-effectiveness of early rehabilitation after lumbar disc surgery was compared to no referral [18]. For the current study, utility values collected at baseline, 12, and 26 weeks and costs collected at 6, 12, and 26 weeks were used. For all scenarios, mean imputation was used to impute missing values at baseline [5]. Of the 169 participants used in our study, 13% (*n* = 22) had missing cost and/or utility data at one or more follow-up time points (Supplementary material 4). Stepwise backwards regression models with *p* < 0.05, were used to identify baseline variables that were predictive of the missingness of data and/or the cost-effectiveness outcomes. The identified variables were added to the imputation model as auxiliary variables (i.e. age, level of education, utility values, Oswestry Disability Index [ODI], pain intensity, Örebro Musculoskeletal Pain Screening Questionnaire [OMPSQ], and the credibility and expectancy surgery [CEQ]) [18]. Missing cost and utility data were imputed using multivariate imputation by chained equations (MICE; FCS-standard) [33] with PMM, stratified by treatment group [34]. Ten datasets were imputed to guarantee a loss of efficiency < 0.05. SUR and LLM were then fitted to the imputed data. The LMM models (i.e., M-LLM and MI-LLM) and SUR models (i.e., SUR-CCA, M-SUR, and MI-SUR) did not include auxiliary variables, and only were corrected for confounders (i.e. baseline utility values, ODI, OMPSQ, and CEQ). The LLM analysis model included both, auxiliary variables and confounders as, in doing so, it should lead to similar results when compared to MI-LL [5] (Supplementary material 4).

In the second trial (empirical dataset 2), the cost-effectiveness of an interpersonal psychotherapy for older adults with major depression was compared to care as usual (i.e., control). For this study, utility values collected at baseline, 6, and 12 months and costs collected at 2, 6, and 12 months were used [19]. Mean imputation was used to impute missing values at baseline [5]. Of the 143 participants, 68% (*n* = 98) of cost and utility data were missing at one or more follow-up time points (Supplementary material 4). Stepwise backwards regression models with *p* < 0.05, were used to identify baseline variables that were predictive of the missingness of data and/or the cost-effectiveness outcomes. The identified variables were added to the imputation model as auxiliary variables (i.e., age, activity daily living [ADL], utility values, alcohol-induced disorder, and mental health problems utility values, marital status, and household composition) [19]. Missing cost and utility data were imputed using multivariate imputation by chained equations (MICE; FCS-standard) [33] with PMM by treatment group[34]. Twenty datasets were imputed to guarantee a loss of efficiency < 0.05. SUR and LLM were then fitted to the imputed data. The LMM models (i.e., M-LLM and MI-LLM) and SUR models (i.e., SUR-CCA, M-SUR, and MI-SUR) did not include auxiliary variables and only were corrected for confounders (i.e., baseline utility values, marital status, and household composition). The LLM analysis model included both, auxiliary variables and confounders [5] (Supplementary material 4).

#### Cost-effectiveness analysis of the empirical datasets

Cost-effectiveness analyses were performed using the differences in costs and QALY between treatment groups estimated by all of the methodological strategies described under 2.2.1. to both empirical datasets. Cost-effectiveness acceptability curves (CEACs) were estimated using the parametric Incremental Net Benefit (INB) approach, where the estimate of acceptability was obtained as the probability that INB > 0 for every value of the willingness-to-pay threshold (WTP) [39, 40]. The INB is defined as $INB=\lambda \times {\Delta }_{q}-{\Delta }_{c}$ and the probability (Pr) of the INB being positive conditional to $\lambda$ is estimated as:

7 $$Pr\left(INB>0|\lambda \right)=1-\Phi \left(\frac{\lambda \times { \Delta }_{q} - {\Delta }_{c}}{\sqrt{({\lambda }^{2} \times Var({ \Delta }_{q})+Var\left({\Delta }_{c}\right) - 2 \times \lambda \times Cov({ \Delta }_{q},{\Delta }_{c})}}\right)$$

where λ is the WTP, $\Phi$ is the cumulative standard normal distribution, and ${\Delta }_{q}$, ${\Delta }_{c}$ are the differences in QALY and total costs between the intervention and control, respectively. $Var\left({\Delta }_{q}\right), Var\left({\Delta }_{c}\right)$, are the variances around differences in QALY and total costs, and $Cov({ \Delta }_{q},{\Delta }_{c})$ is the covariance. To facilitate interpretation of the results, the probability of cost-effectiveness was also reported for willingness-to-pay thresholds of 10,000, 20,000 and 50,000 € per QALY gained [41].

## Results

### Comparison of methodological strategies

#### Simulated datasets

For costs, LLM, MI-LLM and MI-SUR resulted in lower EBs and RMSEs compared to M-LLM, SUR-CCA, and M-SUR for all proportions of missing data. For 10 and 25% of missing data, MI-LLM and MI-SUR resulted in lower EBs and RMSEs compared to LLM. Moreover, LLM and MI-LLM were associated with relatively high levels of overcoverage for 10% and 25% of missing data, whereas for MI-SUR CR was closest to the nominal value. For 50% of missing data, all methodological strategies resulted in relatively higher EBs and RMSEs compared to those found in 10 and 25% of missing data. For LLM, CR were closest to the nominal value compared to the other methodological strategies at 50% of missing data.

**Fig. 1:**
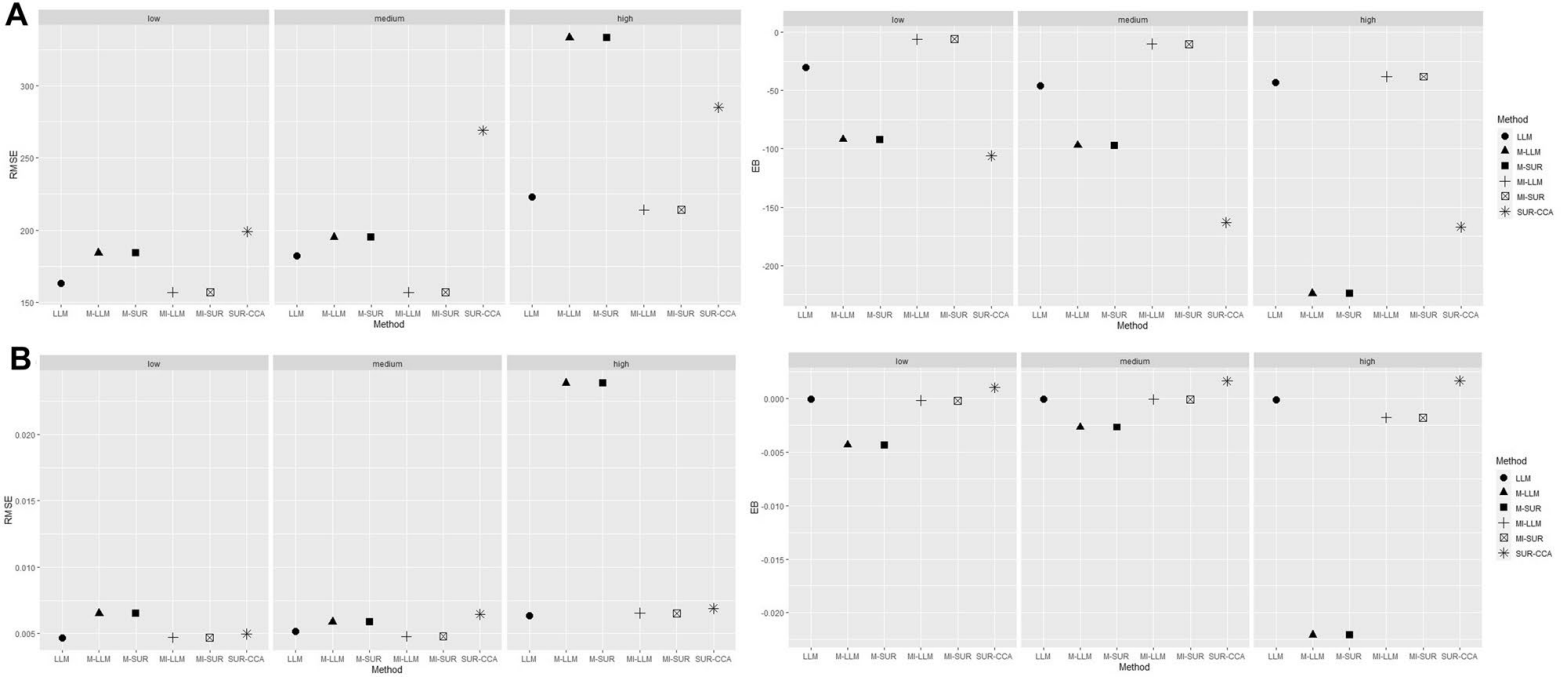
Plots showing root-mean-square error (RMSE) and empirical bias (EB) associated with the six methodological strategies (Method) at different proportions of missing data (i.e., low: 10%, medium: 25%, and high: 50%) for costs (**A**) and QALY (**B**). QALY: quality-adjusted life-year. LLM: longitudinal mixed-model. M-LLM: Mean imputation combined with LLM. MI-LLM: Multiple imputation combined with LLM. SUR-CCA: Seemingly unrelated regressions—complete case analysis**.** M-SUR: mean imputation combined with SUR. MI-SUR: multiple imputation combined with SUR

For QALY, LLM, MI-LLM, and MI-SUR resulted in lower EBs and RMSEs compared to M-LLM, SUR-CCA, and M-SUR for all proportions of missing data. For LLM, EBs and RMSEs were similar or slightly lower than for MI-LLM and MI-SUR for all proportions of missing data. For 10 and 25% of missing data, LLM and MI-LLM were associated with small levels of overcoverage, while MI-SUR was associated with small levels of undercoverage. For 50% of missing data, LLM, MI-LLM, and MI-SUR presented small levels of undercoverage (Table 2, Fig. 1). For both, costs and QALY outcomes, LLM, MI-LLM, and MI-SUR has similar performance measure compared to the LLM and SUR models based on complete datasets.

**Table 2:** Performance measures of the methodological strategies for Costs and QALY

	Costs, €	LLM	M-LLM	MI-LLM	SUR-CCA	M-SUR	MI-SUR
Complete data	EB (MCse)	0.19 (3)	NA	NA	0.17 (3)	NA	NA
	RMSE (MCse)	153 (27)	NA	NA	153 (27)	NA	NA
	CR (MCse)	0.995 (0.158)	NA	NA	0.957 (0.456)	NA	NA
Missing 10%	EB (MCse)	− 30 (4)	− 92 (4)	− 6 (3)	− 106 (4)	− 92 (4)	− 6 (3)
	RMSE (MCse)	164 (29)	184 (32)	157 (27)	199 (34)	184 (32)	157 (27)
	CR (MCse)	0.993 (0.180)	0.981 (0.305)	0.993 (0.180)	0.913 (0.630)	0.895 (0.685)	0.948 (0.496)
Missing 25%	EB (MCse)	− 46 (4)	− 97 (4)	− 10 (3)	− 163 (6)	− 97 (4)	− 10 (3)
	RMSE (MCse)	182 (32)	195 (34)	157 (27)	269 (47)	195 (34)	157 (27)
	CR (MCse)	0.987 (0.253)	0.966 (0.402)	0.996 (0.141)	0.892 (0.694)	0.855 (0.787)	0.951 (0.483)
Missing 50%	EB (MCse)	− 43 (5)	− 224 (7)	− 38 (5)	− 167 (6)	− 224 (7)	− 38 (5)
	RMSE (MCse)	223 (40)	333 (56)	214 (38)	285 (49)	333 (56)	214 (38)
	CR (MCse)	0.950 (0.485)	0.671 (1.050)	0.972 (0.368)	0.860 (0.776)	0.507 (1.118)	0.925 (0.589)
	QALY	LLM	M-LLM	MI-LLM	SUR-CCA	M-SUR	MI-SUR
Complete data	EB (MCse)	− 0.0000589 (0.0001004)	NA	NA	− 0.0000586 (0.0001004)	NA	NA
	RMSE (MCse)	0.0044895 (.0008193)	NA	NA	0.0044890 (0.0008194)	NA	NA
	CR (MCse)	0.914 (0.625)	NA	NA	0.948 (0.496)	NA	NA
Missing 10%	EB (MCse)	− 0.0000687 (0.0001050)	− 0.0043361 (0.0001455)	− 0.0001805 (0.0001053)	0.0010317 (0.0001113)	− 0.0043359 (0.0001455)	− 0.0001803 (0.0001053)
	RMSE (MCse)	0.0046931 (0.0008550)	0.0065054 (0.0011015)	0.0047081 (0.0008567)	0.0049750 (0.0009084)	0.0065048 (0.0011014)	0.0047078 (0.0008568)
	CR (MCse)	0.975 (0.346)	0.906 (0.651)	976 (0.342)	0.951 (0.483)	0.847 (0.804)	0.925 (0.476)
Missing 25%	EB (MCse)	− 0.0000731 (0.0001151)	− 0.0026774 (0.0001314)	− 0.0000688 (0.0001071)	0.0016466 (0.0001443)	− 0.0026772 (0.0001314)	− 0.0000684 (0.0001071)
	RMSE (MCse)	0.0051474 (0.0009316)	0.0058771 (0.0010395)	0.0047891 (0.0008516)	0.0064497 (0.0011593)	0.0058766 (0.0010395)	0.0047889 (0.0008516)
	CR (MCse)	0.966 (0.402)	0.934 (0.555)	0.978 (0.328)	0.950 (0.487)	0.860 (0.774)	0.944 (0.514)
Missing 50%	EB (MCse)	− 0.0001218 (0.0001422)	− 0.0220931 (0.0005334)	− 0.0017733 (0.0001462)	0.00167082 (0.0001542)	− 0.0220928 (0.0005334)	− 0.00177395 (0.0001462)
	RMSE (MCse)	0.0063599 (0.0011411)	0.0238472 (0.0030351)	0.0065377 (0.0011707)	0.0068928 (0.0012258)	0.0238469 (0.0030351)	0.00653833 (0.0011710)
	CR (MCse)	0.936 (0.545)	0.095 (0.657)	0.947 (0.501	0.932 (0.561)	0.085 (0.625)	0.926 (0.583)

*LLM* longitudinal mixed-model, *M-LLM* Mean imputation combined with LLM, *MI-LLM* Multiple imputation combined with LLM, *SUR-CCA* Seemingly unrelated regressions–complete case analysis, *M-SUR* mean imputation combined with SUR, *MI-SUR* multiple imputation combined with SUR, *MCse* Monte Carlo standard error, *EB* empirical bias, *RMSE* root-mean-square error, *CR* coverage rate, *QALY* quality-adjusted life-year, €: Euros

#### Empirical datasets

**Fig. 2:**
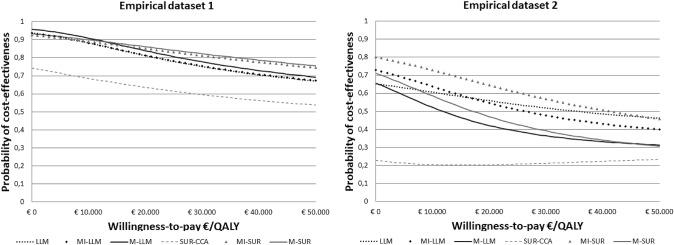
Cost-effectiveness acceptability curves (CEACs) showing the probability of the intervention being cost-effective (x-axis) for different willingness-to-pay thresholds per unit of quality-adjusted life-year (QALY) gained (y-axis) in empirical datasets 1 and 2 with 9% and 53% of missing data in costs and QALY, respectively. LLM: longitudinal mixed-model. M-LLM: Mean imputation combined with LLM. MI-LLM: Multiple imputation combined with LLM. SUR-CCA: Seemingly unrelated regressions—complete case analysis**.** M-SUR: mean imputation combined with SUR. MI-SUR: multiple imputation combined with SUR. €: Euros

In empirical dataset 1, LLM, MI-LLM, and MI-SUR resulted in similar point estimates for QALYs, but not for costs. The 95%CIs around the cost and QALY differences were somewhat wider for LLM and MI-LLM compared to MI-SUR, but all 95%CIs showed that both differences were not statistically significant. Similar ICERs and probabilities of cost-effectiveness were found for LLM, MI-LLM, and MI-SUR. The mean imputation methods (M-LLM and M-SUR) resulted in narrower 95%CIs compared to LLM MI-LLM, and MI-SUR, but similar probabilities of cost-effectiveness. SUR-CCA resulted in very different point estimates, ICER, and probabilities of cost-effectiveness compared to all others methodological strategies (Table 3; Fig. 2).

**Table 3:** Cost-effectiveness results for different proportions of missing data in empirical datasets

Missing	Method	∆ Costs, € (95% CI)	∆ QALY (95% CI)	ICER, €/QALY	Probability of cost-effectiveness			
					€0/ QALY	€10,000/ QALY	€20,000/ QALY	€50,000/ QALY
Empirical dataset 1, *N* = 169								
13%	LLM	− 1048 (− 2391; 296)	− 0.002 (− 0.072; 0.068)	441,749	0.937	0.882	0.812	0.671
	M-LLM	− 1073 (− 2290; 143)	− 0.001 (− 0.069; 0.066)	762,552	0.958	0.909	0.839	0.693
	MI-LLM	− 1046 (− 2391; 298)	− 0.002 (− 0.072; 0.068)	536,379	0.936	0.882	0.813	0.674
	SUR-CCA, *N* = 141	− 332 (− 1324; 660)	− 0.005 (− 0.029; 0.020)	70,233	0.744	0.686	0.636	0.538
	M-SUR	− 681 (− 1569; 207)	− 0.001 (− 0.023; 0.020)	489,833	0.934	0.900	0.861	0.755
	MI-SUR	− 711 (− 1674; 252)	− 0.002 (− 0.025; 0.022)	437,230	0.926	0.891	0.851	0.746
Empirical dataset 2, *N* = 143								
66%	LLM	− 630 (− 3773; 2513)	− 0.018 (− 0.103; 0.067)	35,272	0.653	0.606	0.559	0.462
	M-LLM	− 356 (− 2089; 1376)	− 0.030 (− 0.115; 0.055)	11,785	0.657	0.523	0.421	0.312
	MI-LLM	− 816 (− 3431; 1798)	− 0.031 (− 0.129; 0.067)	26,242	0.730	0.638	0.546	0.398
	SUR-CCA, *N* = 45	798 (− 1299; 2894)	− 0.017 (− 0.090; 0.057)	− 47,329	0.228	0.204	0.203	0.233
	M-SUR	− 309 (− 1395; 777)	− 0.018 (− 0.058; 0.021)	17,132	0.717	0.585	0.470	0.307
	MI-SUR	− 763 (− 2533; 1008)	− 0.018 (− 0.067; 0.030)	41,151	0.800	0.729	0.646	0.458

*LLM* longitudinal mixed-model. *M-LLM* Mean imputation combined with LLM. *MI-LLM *Multiple imputation combined with LLM. *SUR-CCA* Seemingly unrelated regressions–complete case analysis. *M-SUR* mean imputation combined with SUR, *MI-SUR* multiple imputation combined with SUR, ∆ difference, *CI* confidence interval, *ICER* incremental cost-effectiveness ratio, *QALY* quality-adjusted life-year, € Euros

In empirical dataset 2, as in empirical dataset 1, similar point estimates for QALY, but not for costs were found for LLM, MI-LLM, and MI-SUR. Results of SUR-CCA, M-SUR, and M-LLM were most different from those of LLM, MI-LL, and MI-SUR (Table 3). Figure 2 shows that the probabilities of cost-effectiveness of the LLM, M-LLM, MI-LLM, M-SUR, and MI-SUR somewhat differed, particularly at lower WTP thresholds.

## Discussion

### Main findings

Our findings suggest that MI prior to LLM does improve the method’s performance for costs. However, at 50% missing data, all methods had a relatively high level of bias for costs. For QALY, LLM alone already has an acceptable level of performance. Furthermore, the performance of MI-LLM and MI-SUR was similar for costs as well as QALY. Finally, LLM, MI-LLM, and MI-SUR were found to perform considerably better than both mean imputation approaches (i.e., M-LLM, M-SUR) and SUR-CCA for costs and QALY. In empirical datasets, we found LLM, MI-LLM, and MI-SUR to result in similar point estimates for QALY, but not for costs, while the results of SUR-CCA, M-SUR and M-LLM were extensively different.

### Interpretation of findings and comparison with the literature

Previous studies suggest that when using LLM, MI of missing values is not necessary to obtain unbiased effect estimates regardless of the missing data mechanism [14, 15]. In line with these studies, we found MI-LLM and LLM to perform equally well for QALY [14, 15]. For costs, however, we found MI prior to LLM to perform better than LLM alone. This difference in performance is likely because costs are typically associated with higher levels of skewness, kurtosis, within-subject variability, and between-subject variability compared with QALY. This phenomenon was also observed in our simulated as well as our empirical datasets. The LLM Stata command we used in our study assumes multivariate normality, whereas the PMM method used to impute data assumes that the distribution of missing values is the same as the observed one, which may result in more robustness to normality violations than maximum likelihood-based methods. According to Dong et al. (2013), this is so because violation of the multivariate normality assumption may cause convergence problems for maximum likelihood-based methods, while the posterior distribution in MI is approximated by a finite mixture of the normal distributions, enabling MI to capture non-normal features (e.g., skewness) [42]. To the best of our knowledge, however, systematic comparisons of MI and maximum likelihood-based methods in terms of their sensitivity to the violation of the multivariate normality assumption are lacking [42–44]. Recently, Gabrio et al. [31] assessed the applicability of LLM in one empirical trial-based economic evaluation showing similar point estimates for costs and QALY between LLM and MI-LLM [31]. In another study, Gabrio et al. [28] showed that a longitudinal model alone resulted in unbiased estimates under MAR in the context of a trial-based economic evaluation. Gabrio et al. [28], however, considered Bayesian joint longitudinal models, rather than mixed effects models, and did not assess their methods’ performance for costs and QALY separately [28]. In contrast to Gabrio et al. [28] we did not consider any correlation between costs and QALY in our maximum likelihood-based models, whereas costs and effects are typically correlated in trial-based economic evaluations. In a post-hoc analysis we, therefore, assessed whether our maximum likelihood-based models would perform better when specifying them according to the suggestion of Faria et al. (2014). That is, after rescaling costs to the same scale as utility values (i.e. 0–1), both outcomes were stacked on top of each other and simultaneously regressed upon the various covariates in the model using a three-level structure (i.e. subject, outcome, time)(see Supplementary material 5) [5]. However, even though we did find that such a joint estimation of total costs and QALY slightly improved the models’ bias, their coverage rates were found to be highly sensitive to an incorrect rescaling of costs, which makes the approach hard to apply in practice (Supplementary material 5). We also found MI-LLM to perform equally well as MI-SUR, while the former does not consider the possible correlation between costs and QALY and the latter does. This is in line with the results of Mutubuki et al. [45] who found accounting for the correlation between costs and effects not to have a large impact on cost and QALY estimates in two empirical datasets, nor on the statistical uncertainty surrounding both outcomes [45].

### Strengths and limitations

To the best of our knowledge, our study is the first to assess whether it is necessary to perform MI before LLM to account for missing data in trial-based economic evaluations. Another strength of this study was the use of simulated data, which means that we know the ‘true’ outcomes and can, therefore, assess the statistical performance of the methods. In addition, we calculated the number of simulated datasets needed to draw valid conclusions and the simulated datasets resembled empirical data as closely as possible [30].

This study also has some limitations. First, as previously discussed, LLM assumes multivariate normality, an assumption that is typically violated for costs. Future research should therefore assess whether Bayesian joint longitudinal models are better suited for analyzing longitudinal trial-based economic evaluation data than the methods assessed in our study [28]. This might be because Bayesian models enable the joint estimation of costs and effects, while also allowing the use of different distributions for both outcomes (e.g. Gamma for costs and Beta for QALY) [28, 46]. Second, generating a strong MAR mechanism in simulated datasets with a longitudinal structure is relatively complex because a modification in one parameter impacts on all other parameters. To partially overcome this issue, slight baseline imbalances were introduced for age and gender. This, however, is in contrast with the theoretical advantage of RCTs, namely that the randomization of participants allows researchers the confidence that–on average–treatment groups are similar and that the only difference between both groups is the intervention to be assessed for its (cost-)effectiveness [47]. Nonetheless, every individual trial–by definition–exhibits some form of imbalance with respect to measured prognostic variables, especially smaller trials. Moreover, when generating the imbalances we made sure that they were small and in line with the slight baseline imbalances encountered in our empirical datasets [47]. Third, LLM can only deal with missing values at the aggregate level (i.e., total costs and utility values), which might make it less suitable when values are missing at the item level (e.g., number of GP visits, EQ-5D mobility dimension). Further research is therefore needed to assess whether the current results would hold when data are missing at the item-level. Fourth, four follow-up time points were simulated with equal time intervals (i.e., 3, 6, 9, and 12-months follow-up), whereas this is not necessarily the case in trial-based economic evaluations [48]. Further research is needed to assess the impact of varying time intervals on the performance of the methods assessed in this study. Fifth, in this study, we looked at MAR conditional on baseline values, whereas MAR can also be conditional on other values in the dataset. However, we do not expect our conclusion to change for MAR conditional at other values, because such other values can easily be included in an imputation model as well. Sixth, another limitation of the study is that results have only been assessed under MAR mechanisms, while MNAR is also possible. Since it is never possible to distinguish between MAR and MNAR from the data at hand, the robustness of deviations from the MAR assumption is ideally assessed in trial-based economic evaluations using sensitivity analyses. In doing so, the magnitude and direction of the departures from MAR are ideally defined based on external information (e.g. expert opinion) [5, 7]. Seventh, it is also relevant to mention that despite simulating skewed cost and effect data, we assumed that with the current sample size (i.e. 600 subjects), LMM estimates would be robust to non-normal distribution [49]. Amongst others, we did so because using bootstrapping to deal with skewed data made our simulations slower and time consuming. In addition, we wanted to ensure that the differences in performance could be attributed to the use of MI and/or LLM, and not to bootstrapping, and consensus does not currently exist as to how MI ought to be combined with bootstrapping [50, 51].

### Implications for practice and research

MI-LLM was found to perform better than LLM in the simulated datasets and equally well as MI-SUR. Therefore, if researchers want to use LLM for analyzing trial-based economic evaluation data they are advised to multiply impute missing values first. Next to the identified improved performance of LLM, MI also allows to include values in the imputation model that may be not relevant for the analysis model (i.e., auxiliary variables), allows to impute missing values at the item-level, and allows for a wider range of regression models to be used in the analysis (e.g., SUR) [23]. From an efficiency standpoint, one might also opt for MI-SUR instead of MI-LLM, because it is computationally more efficient, while having similar EBs and RMSEs and CRs that are closer to the nominal value. Nevertheless, one should bear in mind, that SUR does not allow for the estimation of the average intervention effect over time, whereas LLM does. On the other hand, SUR account for the correlation between costs and effects, whereas LLM does not. Another important point is that variables included in the LLM model should have complete data at baseline otherwise, the model will exclude missing observations from the analysis. To facilitate researchers in using either one of these methods, software codes are included to this manuscript as Supplementary materials 3, and 5. Moreover, as the methods’ performance was found to be considerably less with 50% missing data, researchers are advised to extensively assess the robustness of their results to the methods applied for handling missing data, particularly with high percentages of missing data.

As previously discussed, it should be noted that the presence of item-level missingness highly depends on the measures used to collect the data. For example, it is rarely the case to observe item-level missingness in short questionnaires, such as the EQ-5D, which almost always exhibits monotone patterns [29]. On the contrary, resource use questionnaires often present item-level missingness in trial-based economic evaluations, which makes non-monotone pattern likely to be more relevant for costs. In the current study, we only simulated monotone patterns, hence further investigation is needed to assess whether the current results hold when data are missing at the item level and/or when time intervals vary over time.

Further research is also needed to assess whether Bayesian joint longitudinal models are preferred over the frequentist models evaluated in the current study. Among others, the relevance of exploring the performance of Bayesian models are that they can encode missingness assumptions via prior distributions (e.g., MNAR) and quantify uncertainty without the need to implement bootstrapping methods [52, 53].

## Conclusion

Our findings suggest that if researchers want to use LLM for analyzing trial-based economic evaluation data, it is advisable to multiply impute missing values first. One might also opt for a combination of MI and SUR. MI-SUR is computationally more efficient than MI-LLM, but it does not allow for the estimation of average intervention effects over time while having the advantage of accounting for the correlation between costs and effects. Our findings also underscore the importance of extensively assessing the robustness of results to the methods applied for handling missing data, particularly with high percentages of missing data. Further research should assess the relative performance of MI-LLM and MI-SUR versus Bayesian joint longitudinal models, under MAR and MNAR assumptions and assess whether the current results hold when data are missing at the item-level and/or when time intervals vary over time.

## Supplementary Information

Below is the link to the electronic supplementary material.

Supplementary file1 (DOCX 34 KB)

Supplementary file2 (DOCX 18 KB)

Supplementary file3 (DOCX 30 KB)

Supplementary file4 (DOCX 31 KB)

Supplementary file5 (DOCX 168 KB)
