# Supplementary material for: The handling of missing data in trial-based economic evaluations: should data be multiply imputed prior to longitudinal linear mixed-model analyses?
Source: Eur J Health Econ. 2022 Sep 26;24(6):951–65. doi: 10.1007/s10198-022-01525-y (PMC10290620; doi:10.1007/s10198-022-01525-y)
Supplement: Supplementary file 1 — Supplementary file1 (DOCX 34 KB) [file 10198_2022_1525_MOESM1_ESM.docx]

**SUPPLEMENTARY MATERIAL 5**

**Joint Longitudinal Linear Mixed-model analysis –**  In this strategy, a joint LLM for costs and utility values was specified according to Faria et al. (2014), instead of two separated LLM models. That is, after rescaling costs to the same scale as utility values (i.e. 0-1), both outcomes were stacked on top of each other and simultaneously regressed upon the various covariates in the model using a three-level structure (i.e. subject, outcome, time) (please see Stata code in page 2). Results are presented in the table below.

**Supplementary Table 3. Performance of the joint longitudinal linear mixed-models for Costs and QALY**

|  | **Costs,** € | **JOINT-LLM** | **M-JOINT-LLM** | **MI-LLM** | **SUR-CCA** | **M-SUR** | **MI-SUR** |
| --- | --- | --- | --- | --- | --- | --- | --- |
| **Complete data** | EB (MCse) | 0.19 (3) | NA | NA | 0.17 (3) | NA | NA |
|  | RMSE (MCse) | 153 (27) | NA | NA | 153 (27) | NA | NA |
|  | CR (MCse) | 0.995 (0.158) | NA | NA | 0.957 (0.456) | NA | NA |
| **Missing**  **10%** | EB (MCse) | -30 (4) | -92 (4) | -6 (3) | -106 (4) | -92 (4) | -6 (3) |
|  | RMSE (MCse) | 164 (29) | 184 (32) | 157 (27) | 199 (34) | 184 (32) | 157 (27) |
|  | CR (MCse) | 0.939 (0.533) | 0.898 (0.675) | 0.948 (0.496) | 0.913 (0.630) | 0.895 (0.685) | 0.948 (0.496) |
| **Missing**  **25%** | EB (MCse) | -41 (4) | -97 (4) | -10 (3) | -163 (6) | -97 (4) | -10 (3) |
|  | RMSE (MCse) | 182 (32) | 194 (34) | 157 (27) | 269 (47) | 195 (34) | 157 (27) |
|  | CR (MCse) | 0.941 (0.527) | 0.866 (0.760) | 0.955 (0.463) | 0.892 (0.694) | 0.855 (0.787) | 0.951 (0.483) |
| **Missing**  **50%** | EB (MCse) | -43 (5) | -224 (7) | -38 (5) | -167 (6) | -224 (7) | -38 (5) |
|  | RMSE (MCse) | 223 (40) | 333 (56) | 214 (38) | 285 (49) | 333 (56) | 214 (38) |
|  | CR (MCse) | 0.905 (0.656) | 0.583 (1.115) | 0.933 (0.560) | 0.860 (0.776) | 0.507 (1.118) | 0.925 (0.589) |
|  | **QALY** | **JOINT-LLM** | **M-JOINT-LLM** | **MI-LLM** | **SUR-CCA** | **M-SUR** | **MI-SUR** |
| **Complete data** | EB (MCse) | -0.0000589  (0.0001004) | NA | NA | -0.0000586  (.0001004) | NA | NA |
|  | RMSE (MCse) | .0044895  (.0008193) | NA | NA | 0.0044890  (0.0008194) | NA | NA |
|  | CR (MCse) | 0.914 (0.625) | NA | NA | 0.948 (0.496) | NA | NA |
| **Missing**  **10%** | EB  (MCse) | -0.00000736 (0.0001051) | -0.004345 (0.0001457) | -0.000184 (0.0001054) | 0.0010317 (0.0001113) | -0.0043359 (0.0001455) | -0.0001803 (0.0001053) |
|  | RMSE  (MCse) | 0.0046984 (0.0008556) | 0.0065157 (0.0011049) | 0.0047126 (0.0008573) | 0.0049750 (0.0009084) | 0.0065048 (0.0011014) | 0.0047078 (0.0008568) |
|  | CR (MCse) | 0.899 (0.674) | 0.729 (0.993) | 0.907 (0.649) | 0.951 (0.483) | 0.847 (0.804) | 0.925 (0.476) |
| **Missing**  **25%** | EB  (MCse) | -0.0000763 (0.0001153) | -0.0026896 (0.0001318) | -0.0000729 (0.0001072) | 0.0016466 (0.0001443) | -0.0026772 (0.0001314) | -0.0000684 (0.0001071) |
|  | RMSE  (MCse) | 0.0051572 (0.0009329) | 0.0058925 (0.0010421) | 0.0047946 (0.0008518) | 0.0064497 (0.0011593) | 0.0058766 (0.0010395) | 0.0047889 (0.0008516) |
|  | CR (MCse) | 0.903 (0.662) | 0.791 (0.909) | 0.904 (0.659) | 0.950 (0.487) | 0.860 (0.774) | 0.944 (0.514) |
| **Missing**  **50%** | EB  (MCse) | -0.0001245 (0.0001424) | -0.02207 (0.0005329) | -0.0001778 (0.0001463) | 0.00167082 (0.0001542) | -0.0220928 (0.0005334) | -0.00177395 (0.0001462) |
|  | RMSE  (MCse) | 0.00636641 (0.0011428) | 0.023827 (0.0030349) | 0.0065429 (0.0011727) | 0.0068928 (0.0012258) | 0.0238469 (0.0030351) | 0.00653833 (0.0011710) |
|  | CR (MCse) | 0.865 (0.764) | 0.045 (0.463) | 0.875 (0.738) | 0.932 (0.561) | 0.085 (0.625) | 0.926 (0.583) |

JOINT-LLM: joint longitudinal linear mixed-model (i.e., costs and utilities at each time point were simultaneously regressed upon the various covariates in the model after rescaling their values). M-JOINT-LLM: Mean imputation combined with JOINT-LLM. MI-JOINT-LLM: Multiple imputation combined with JOINT-LLM. SUR-CCA: Seemingly unrelated regressions - complete case analysis**.** M-SUR: mean imputation combined with SUR. MI-SUR: multiple imputation combined with SUR. MCse: Monte Carlo standard error. EB: empirical bias. RMSE: root-mean-square error. CR: coverage rate. QALY: quality-adjusted life-year. €: Euros.

**/* Stata code - JOINT LONGITUDINAL LINEAR MIXED-MODEL */**

clear

set more off

cd "C:\MISSINGX"

local n = 2000

forvalues j = 1(1)`n' {

local y = `j'

use "dataset`y'", clear

//costs are scaled down by 7000 to transform them into a similar scale as utility values

replace cT0 = cT0/7000

replace cT1 = cT1/7000

replace cT2 = cT2/7000

replace cT3 = cT3/7000

replace cT4 = cT4/7000

//keep baseline values for costs and utilities in wide format

gen utility_b = uT0

gen cost_b = cT0

//reshape from wide to long creating a new variable - time - that indicates time period

reshape long cT uT, i(id) j(time 0 1 2 3 4)

rename cT cost

rename uT utility

rename cost y1

rename utility y2

//reshape again to create a single dependent variable - y. The variable type indicates whether it refers to costs or utilities

reshape long y, i(id time) j(type)

gen cost=type==1

gen QALY=type==2

egen timetype=group(time type)

//JOINT-LLM

quietly mixed y i.cost#i.time i.cost#i.trt#i.time i.cost#i.time#c.utility_b i.cost#i.time#c.cost_b i.cost#i.time#c.age i.cost#i.time#i.gender || id:

mat betaCE = e(b) /* extract matrix of betas */

mat vari = e(V) /* extract matrix of variances */

mat obs = e(N_g) /* extract number of observations used in the model */

gen obs = obs[1,1]

gen Za = 1.95996

/*i.cost#i.year represents the interaction between the cost and utilities and each time point;

i.cost#i.trt#i.time represents the effect of treatment (trt) on costs and utilities at each time point;

i.cost#i.time#c.utility_b represents the effect of utility at baseline on costs and utilities at each time point.

i.cost#i.time#c.cost_b represents the effect of cost at baseline on costs and utilities at each time point. */

//gen variables for utility difference at each time point adjusted for baseline costs

gen utility_diff1 = betaCE[1,17]

gen utility_diff2 = betaCE[1,18]

gen utility_diff3 = betaCE[1,19]

gen utility_diff4 = betaCE[1,20]

//calculate QALY difference

gen QALY_diff = (0.5*(utility_diff1+utility_diff1)*(3/12)) + (0.5*(utility_diff1+utility_diff2)*(3/12)) + (0.5*(utility_diff2+utility_diff3)*(3/12)) + (0.5*(utility_diff3+utility_diff4)*(3/12))

//gen variables for variance utility difference at each time point

gen varu1 = vari[17,17]

gen varu2 = vari[18,18]

gen varu3 = vari[19,19]

gen varu4 = vari[20,20]

//calculate variance of QALY difference

gen QALY_var = ((0.25*(varu1+varu1)) + (0.25*(varu1+varu2)) + (0.25*(varu2+varu3)) + (0.25*(varu3+varu4)))/4

//calculate SE of QALY difference

gen SE_QALY_diff = sqrt(QALY_var)

//estimate CI around QALY difference

gen LL_QALY = QALY_diff - Za*SE_QALY_diff

gen UL_QALY = QALY_diff + Za*SE_QALY_diff

//gen variables for cost difference at each time point adjusted for baseline costs

gen cost_diff1 = betaCE[1,27]

gen cost_diff2 = betaCE[1,28]

gen cost_diff3 = betaCE[1,29]

gen cost_diff4 = betaCE[1,30]

//calculate marginal cost difference

gen cost_diff = (cost_diff1 + cost_diff2 + cost_diff3 + cost_diff4)*7000

//gen variables for variance of cost difference at each time point

gen varc1 = vari[27,27]

gen varc2 = vari[28,28]

gen varc3 = vari[29,29]

gen varc4 = vari[30,30]

//calculate SE for marginal cost difference

gen SEc1 = sqrt(varc1)

gen SEc2 = sqrt(varc2)

gen SEc3 = sqrt(varc3)

gen SEc4 = sqrt(varc4)

gen SE_cost_diff = (SEc1 + SEc2 + SEc3 + SEc4)*7000

//estimate CI around marginal cost difference

gen LL_costs = (cost_diff - Za*SE_cost_diff)

gen UL_costs = (cost_diff + Za*SE_cost_diff)

//calculate covariance costs and QALY

gen cov = vari[2,17]*7000

save "JOINT-LLM\postboots`y'", replace

}

**/* MULTIPLE IMPUTATION + JOINT LONGITUDINAL LINEAR MIXED-MODEL*/**

clear

set more off

cd "C:\MISSINGX"

local n = 2000

forvalues j = 1(1)`n' {

local y = `j'

use "dataset`y'", clear

// MULTIPLE IMPUTATION MODEL

mi set flong

mi register regular age gender trt uT0 cT0

mi register imputed cT1 cT2 cT3 cT4 uT1 uT2 uT3 uT4

quietly mi impute chained (pmm, knn(5)) cT1 cT2 cT3 cT4 uT1 uT2 uT3 uT4 = age gender uT0 cT0, by(trt) replace add(10) rseed(`k')

save "C:\MISSINGX\MI-JOINT-LLM\dataset`y'_imp", replace

}

clear

set more off

cd "C:\MISSINGX\MI-JOINT-LLM"

local n = 2000

forvalues k=1(1)`n' {

local y = `k'

use "dataset`y'_imp", clear

//costs are scaled down by 7000 to transform them into a similar scale as utilities

replace cT0 = cT0/7000

replace cT1 = cT1/7000

replace cT2 = cT2/7000

replace cT3 = cT3/7000

replace cT4 = cT4/7000

//keep baseline values for costs and utilities in wide format

gen utility_b = uT0

gen cost_b = cT0

//reshape from wide to long creating a new variable - time - that indicates time period

rename (cT0 cT1 cT2 cT3 cT4) (cost0 cost1 cost2 cost3 cost4)

rename (uT0 uT1 uT2 uT3 uT4) (utility0 utility1 utility2 utility3 utility4)

quietly mi reshape long cost utility, i(id) j(time 0 1 2 3 4)

rename cost y1

rename utility y2

//reshape again to create a single dependent variable - y. The variable type indicates whether it refers to costs or utilities

mi reshape long y, i(id time) j(type)

gen cost=type==1

gen QALY=type==2

egen timetype=group(time type)

//JOINT-LLM performed in each one of the imputed datasets (MI-JOINT-LLM)

quietly mi estimate: mixed y i.cost#i.time i.cost#i.trt#i.time i.cost#i.time#c.utility_b i.cost#i.time#c.cost_b i.cost#i.time#c.age i.cost#i.time#i.gender|| id:

mat betaCE = e(b_mi) /* extract matric of betas */

mat vari = e(V_mi) /* extract matrix of variances */

mat obs = e(N_g_mi) /* extract number of observations used in the model */

gen obs = obs[1,1]

gen loss_eff = e(fmi_max_mi)/e(M_mi) /* calculate loss of efficiency)

gen Za = 1.95996

//gen variables for variance utility difference at each time point

gen utility_diff1 = betaCE[1,17]

gen utility_diff2 = betaCE[1,18]

gen utility_diff3 = betaCE[1,19]

gen utility_diff4 = betaCE[1,20]

//calculate QALY difference

gen QALY_diff = (0.5*(utility_diff1+utility_diff1)*(3/12)) + (0.5*(utility_diff1+utility_diff2)*(3/12)) + (0.5*(utility_diff2+utility_diff3)*(3/12)) + (0.5*(utility_diff3+utility_diff4)*(3/12))

//calculate variance of QALY difference

gen varu1 = vari[17,17]

gen varu2 = vari[18,18]

gen varu3 = vari[19,19]

gen varu4 = vari[20,20]

//calculate variance of QALY difference

gen QALY_var = ((0.25*(varu1+varu1)) + (0.25*(varu1+varu2)) + (0.25*(varu2+varu3)) + (0.25*(varu3+varu4)))/4

//calculate SE of QALY difference

gen SE_QALY_diff = sqrt(QALY_var)

//estimate CI around QALY difference

gen LL_QALY = QALY_diff - Za*SE_QALY_diff

gen UL_QALY = QALY_diff + Za*SE_QALY_diff

//gen variables for cost difference at each time point adjusted for baseline costs

gen cost_diff1 = betaCE[1,27]

gen cost_diff2 = betaCE[1,28]

gen cost_diff3 = betaCE[1,29]

gen cost_diff4 = betaCE[1,30]

//gen variables for variance of cost difference at each time point

gen cost_diff = (cost_diff1 + cost_diff2 + cost_diff3 + cost_diff4)*7000

//gen variables for variance of cost difference at each time point

gen varc1 = vari[27,27]

gen varc2 = vari[28,28]

gen varc3 = vari[29,29]

gen varc4 = vari[30,30]

//calculate SE for marginal cost difference

gen SEc1 = sqrt(varc1)

gen SEc2 = sqrt(varc2)

gen SEc3 = sqrt(varc3)

gen SEc4 = sqrt(varc4)

gen SE_cost_diff = (SEc1 + SEc2 + SEc3 + SEc4)*7000

//estimate CI around marginal cost difference

gen LL_costs = (cost_diff - Za*SE_cost_diff)

gen UL_costs = (cost_diff + Za*SE_cost_diff)

//calculate covariance costs and QALY

gen cov = vari[2,17]*7000

save "postboots`y'", replace

}

/* **MEAN IMPUTATION + JOINT** **LONGITUDINAL LINEAR MIXED-MODEL** */

clear

set more off

cd "C:\MISSING10"

local n = 2000

forvalues k=1(1)`n' {

local y = `k'

use "dataset`y'", clear

// MEAN IMPUTATION BY TREATMENT GROUP

bysort trt: egen mean_uT1 = mean(uT1)

bysort trt: egen mean_uT2 = mean(uT2)

bysort trt: egen mean_uT3 = mean(uT3)

bysort trt: egen mean_uT4 = mean(uT4)

replace uT1 = mean_uT1 if missing(uT1)

replace uT2 = mean_uT2 if missing(uT2)

replace uT3 = mean_uT3 if missing(uT3)

replace uT4 = mean_uT4 if missing(uT4)

bysort trt: egen mean_cT1 = mean(cT1)

bysort trt: egen mean_cT2 = mean(cT2)

bysort trt: egen mean_cT3 = mean(cT3)

bysort trt: egen mean_cT4 = mean(cT4)

replace cT1 = mean_cT1 if missing(cT1)

replace cT2 = mean_cT2 if missing(cT2)

replace cT3 = mean_cT3 if missing(cT3)

replace cT4 = mean_cT4 if missing(cT4)

//costs are scaled down by 7000 to transform them into a similar scale as utilities

replace cT0 = cT0/7000

replace cT1 = cT1/7000

replace cT2 = cT2/7000

replace cT3 = cT3/7000

replace cT4 = cT4/7000

//keep baseline values for costs and utilities in wide format

gen utility_b = uT0

gen cost_b = cT0

//reshape from wide to long creating a new variable - time - that indicates time period

reshape long cT uT, i(id) j(time 0 1 2 3 4)

rename cT cost

rename uT utility

rename cost y1

rename utility y2

//reshape again to create a single dependent variable - y. The variable type indicates whether it refers to costs or utilities

reshape long y, i(id time) j(type)

gen cost=type==1

gen QALY=type==2

egen timetype=group(time type)

//JOINT-LLM performed in the mean imputed data (M-JOINT-LLM)

quietly mixed y i.cost#i.time i.cost#i.trt#i.time i.cost#i.time#c.utility_b i.cost#i.time#c.cost_b i.cost#i.time#c.age i.cost#i.time#i.gender || id:

mat betaCE = e(b) /* extract matrix of betas */

mat vari = e(V) /* extract matrix of variances */

mat obs = e(N_g) /* extract number of observations used in the model */

gen obs = obs[1,1]

gen Za = 1.95996

//gen variables for utility difference at each time point adjusted for baseline costs

gen utility_diff1 = betaCE[1,17]

gen utility_diff2 = betaCE[1,18]

gen utility_diff3 = betaCE[1,19]

gen utility_diff4 = betaCE[1,20]

//calculate QALY difference

gen QALY_diff = (0.5*(utility_diff1+utility_diff1)*(3/12)) + (0.5*(utility_diff1+utility_diff2)*(3/12)) + (0.5*(utility_diff2+utility_diff3)*(3/12)) + (0.5*(utility_diff3+utility_diff4)*(3/12))

//gen variables for variance utility difference at each time point

gen varu1 = vari[17,17]

gen varu2 = vari[18,18]

gen varu3 = vari[19,19]

gen varu4 = vari[20,20]

//calculate variance of QALY difference

gen QALY_var = ((0.25*(varu1+varu1)) + (0.25*(varu1+varu2)) + (0.25*(varu2+varu3)) + (0.25*(varu3+varu4)))/4

//calculate SE of QALY difference

gen SE_QALY_diff = sqrt(QALY_var)

//estimate CI around QALY difference

gen LL_QALY = QALY_diff - Za*SE_QALY_diff

gen UL_QALY = QALY_diff + Za*SE_QALY_diff

//gen variables for cost difference at each time point adjusted for baseline costs

gen cost_diff1 = betaCE[1,27]

gen cost_diff2 = betaCE[1,28]

gen cost_diff3 = betaCE[1,29]

gen cost_diff4 = betaCE[1,30]

//calculate marginal cost difference

gen cost_diff = (cost_diff1 + cost_diff2 + cost_diff3 + cost_diff4)*7000

//gen variables for variance of cost difference at each time point

gen varc1 = vari[27,27]

gen varc2 = vari[28,28]

gen varc3 = vari[29,29]

gen varc4 = vari[30,30]

//calculate SE for marginal cost difference

gen SEc1 = sqrt(varc1)

gen SEc2 = sqrt(varc2)

gen SEc3 = sqrt(varc3)

gen SEc4 = sqrt(varc4)

gen SE_cost_diff = (SEc1 + SEc2 + SEc3 + SEc4)*7000

//estimate CI around marginal cost difference

gen LL_costs = (cost_diff - Za*SE_cost_diff)

gen UL_costs = (cost_diff + Za*SE_cost_diff)

//calculate covariance costs and QALY

gen cov = vari[2,17]*7000

save "M-JOINT-LLM\postboots`y'", replace

}
