# Supplementary material for: The handling of missing data in trial-based economic evaluations: should data be multiply imputed prior to longitudinal linear mixed-model analyses?
Source: Eur J Health Econ. 2022 Sep 26;24(6):951–65. doi: 10.1007/s10198-022-01525-y (PMC10290620; doi:10.1007/s10198-022-01525-y)
Supplement: Supplementary file 2 — Supplementary file2 (DOCX 18 KB) [file 10198_2022_1525_MOESM2_ESM.docx]

**SUPPLEMENTARY MATERIAL 1**

**Data generating code in R**

# _Simulation of longitudinal data for utilities and costs including covariates at baseline_

## COMPLETE DATA

library(simstudy) # required to generate correlated longitudinal data

library(foreign) # required to save data in .dta

for (i in 1:2000){

set.seed(i)

##Generate baseline characteristics

def <- defData(varname = "variance", formula = 0.005, dist = "normal")

def <- defData(def, varname = "gammaDisC", formula = 0.1, dist = "nonrandom")

def <- defData(def, varname = "nMeasurement", formula = 1, dist = "nonrandom", id = "id")

def <- defData(def, varname = "trt", formula = 0.52, dist = "binary", id="id")

data <- genData(600, def)

defb <- defDataAdd(varname = "age", formula = "40 + (5*trt)", variance = 140, dist="normal")

defb <- defDataAdd(defb, varname = "gender", formula = "0.52 + (0.4*trt)", dist = "binary")

data <- addCorFlex(data, defb, rho = 0.0001, corstr = "cs")

data$age <- ifelse(data$age <= 18, 18, data$age)

data$age <- ifelse(data$age >= 99, 99, data$age)

defb <- defDataAdd(varname = "uT0", formula = "0.2 + (0.0045 * age) + (0.025 * gender)", variance = 0.002, dist = "gamma", link = "logit")

defb <- defDataAdd(defb, varname = "cT0", formula = "50 + (15*age) + (100*gender)", variance = 0.15, dist = "gamma", link = "logit")

data <- addCorFlex(data, defb, rho = -0.5)

##Generate follow-up utilities and costs

defc <- defDataAdd(varname = "uT1", formula = "uT0 + (0.04* trt) + (0.002 * age) + (0.01 * gender)", variance = 0.002, dist = "gamma", link = "logit")

defc <- defDataAdd(defc, varname = "cT1", formula = "cT0 + (62.5*trt) + (1*age) + (10*gender)", variance = 0.15, dist = "gamma", link = "logit")

data <- addCorFlex(data, defc, rho = -0.5)

data <- genCluster(data, "id", "nMeasurement", "idcluster")

##Correlation between time points for utilities

Q <- matrix(c(1.0,0.9,0.9,0.9,1.0,0.9,0.9,0.9,1.0), nrow = 3)

data <- addCorGen(dtOld = data, idvar = "id", nvars = 3, corMatrix = Q, dist = "normal", param1 = "uT1", param2 = "variance", cnames = "uT2, uT3, uT4")

##Correlation between time points for costs

C <- matrix(c(1.0,0.7,0.7,0.7,1.0,0.7,0.7,0.7,1.0), nrow = 3)

data <- addCorGen(dtOld = data, idvar = "id", nvars = 3, corMatrix = C, dist = "gamma", param1 = "cT1", param2 = "gammaDisC", cnames = "cT2, cT3, cT4")

write.dta(data, file = paste0("C:/completedata/dataset",i,".dta"))

}

*Note: the correlations and the coefficients set corresponded approximately to the values generated due to relative complexity of the simulated longitudinal data.*
