# Supplementary material for: The handling of missing data in trial-based economic evaluations: should data be multiply imputed prior to longitudinal linear mixed-model analyses?
Source: Eur J Health Econ. 2022 Sep 26;24(6):951–65. doi: 10.1007/s10198-022-01525-y (PMC10290620; doi:10.1007/s10198-022-01525-y)
Supplement: Supplementary file 3 — Supplementary file3 (DOCX 30 KB) [file 10198_2022_1525_MOESM3_ESM.docx]

**SUPPLEMENTARY MATERIAL 2**

To generate follow-up missing values, a missing data indicator $m_{ij}$ was generated for every subject *i* (*i* = 1, …, N=600) at time point *j* (*j* = 1, …, 4) using a binomial distribution and a logit link function to model the linear dependence between the probability of missing data and its predictive variables at baseline (i.e., age, gender, cT0, uT0, trt).

$$m_{ij} \sim Binomial\left( \pi_{mij} \right),$$

$logit\left( \pi_{mij} \right)= \beta_{0}+ \beta_{1}{age}_{i}+ \beta_{2}g{ender}_{i}+ \beta_{3}{uT0}_{i}+ \beta_{4}{cT0}_{i}+ \beta_{5}{trt}_{i}+ \varepsilon_{i},$ (1)

where $\pi_{mij}$ is the probability of a subject *i* having missing data at time point *j*. The intercept ($\beta_{0})$ and coefficients ($\beta_{1,}\beta_{2,}\beta_{3,}\beta_{4,} \beta_{5})$ of covariates were tweaked to generate a plausible MAR assumption and datasets with 10%, 25%, and 50% missings, and $\varepsilon_{i}$ is the error term (Supplementary material 2)[28]. Then, at the respective time point *j*, all complete follow-up cost and utility values were replaced by missing values according to $m_{ij}$. This process was repeated for all consecutive time points amongst the subset of individuals still in the study[28].

**Supplementary Table 1. Parameter values used for generating missing data**

| **Mechanism, missing %** | | **Parameter values** |
| --- | --- | --- |
| MAR, 10% |  | |
| T1 | $if trt=1, \beta_{1}=0.110; \beta_{2}=1; \beta_{3}=0.65; \beta_{4}=0.00027; \beta_{5}=1$ | |
|  | $if trt=0, \beta_{1}=0.110; \beta_{2}=-1; \beta_{3}=-0.65; \beta_{4}=-0.0001; \beta_{5}=1$ | |
| T2 | $if trt=1,\beta_{0}=-0.1; \beta_{1}=0.100; \beta_{2}=1; \beta_{3}=0.65; \beta_{4}=0.00027; \beta_{5}=1$ | |
|  | $if trt=0, {\beta_{0}=-0.1; \beta}_{1}=0.100; \beta_{2}=-1; \beta_{3}=-0.65; \beta_{4}=-0.00027; \beta_{5}=1$ | |
| T3 | $if trt=1,\beta_{0}=-0.2; \beta_{1}=0.90; \beta_{2}=1; \beta_{3}=0.65; \beta_{4}=0.00027; \beta_{5}=1$ | |
|  | $if trt=0, {\beta_{0}=-0.2; \beta}_{1}=0.80; \beta_{2}=-1; \beta_{3}=-0.65; \beta_{4}=-0.0001; \beta_{5}=1$ | |
| T4 | $if trt=1,\beta_{0}=-1.9; \beta_{1}=0.100; \beta_{2}=1; \beta_{3}=0.65; \beta_{4}=0.00027; \beta_{5}=1$ | |
|  | $if trt=0, {\beta_{0}=-1.9; \beta}_{1}=0.50; \beta_{2}=-1; \beta_{3}=-0.65; \beta_{4}=-0.0001; \beta_{5}=1$ | |
| MAR 25% |  | |
| T1 | $if trt=1, \beta_{1}=0.110; \beta_{2}=1; \beta_{3}=0.65; \beta_{4}=0.00027; \beta_{5}=1$ | |
|  | $if trt=0, \beta_{1}=0.110; \beta_{2}=-1; \beta_{3}=-0.65; \beta_{4}=-0.0001; \beta_{5}=1$ | |
| T2 | $if trt=1,\beta_{0}=-0.1; \beta_{1}=0.100; \beta_{2}=1; \beta_{3}=0.65; \beta_{4}=0.00027; \beta_{5}=1$ | |
|  | $if trt=0, {\beta_{0}=-0.1; \beta}_{1}=0.100; \beta_{2}=-1; \beta_{3}=-0.65; \beta_{4}=-0.00027; \beta_{5}=1$ | |
| T3 | $if trt=1,\beta_{0}=-0.1; \beta_{1}=0.100; \beta_{2}=1; \beta_{3}=0.65; \beta_{4}=0.00027; \beta_{5}=1$ | |
|  | $if trt=0, {\beta_{0}=-0.1; \beta}_{1}=0.033; \beta_{2}=-1; \beta_{3}=-0.65; \beta_{4}=-0.0001; \beta_{5}=1$ | |
| T4 | $if trt=1, \beta_{1}=0.300; \beta_{2}=1; \beta_{3}=0.65; \beta_{4}=0.00027; \beta_{5}=1$ | |
|  | $if trt=0,\beta_{1}=-0.300; \beta_{2}=-1; \beta_{3}=-0.65; \beta_{4}=-0.0001; \beta_{5}=1$ | |
| MAR 50% |  | |
| T1 | $if trt=1, \beta_{1}=0.110; \beta_{2}=1; \beta_{3}=0.65; \beta_{4}=0.00027; \beta_{5}=1$ | |
|  | $if trt=0, \beta_{1}=0.110; \beta_{2}=-1; \beta_{3}=-0.65; \beta_{4}=-0.0001; \beta_{5}=1$ | |
| T2 | $if trt=1,\beta_{0}=-0.1; \beta_{1}=0.100; \beta_{2}=1; \beta_{3}=0.65; \beta_{4}=0.00027; \beta_{5}=1$ | |
|  | $if trt=0, {\beta_{0}=-0.1; \beta}_{1}=0.100; \beta_{2}=-1; \beta_{3}=-0.65; \beta_{4}=-0.00027; \beta_{5}=1$ | |
| T3 | $if trt=1,\beta_{0}=-0.1; \beta_{1}=0.100; \beta_{2}=1; \beta_{3}=0.65; \beta_{4}=0.00027; \beta_{5}=1$ | |
|  | $if trt=0, {\beta_{0}=-0.1; \beta}_{1}=0.033; \beta_{2}=-1; \beta_{3}=-0.65; \beta_{4}=-0.0001; \beta_{5}=1$ | |
| T4 | $if trt=1, \beta_{1}=0.300; \beta_{2}=1; \beta_{3}=0.65; \beta_{4}=0.00027; \beta_{5}=1$ | |
|  | $if trt=0,\beta_{1}=-0.300; \beta_{2}=-1; \beta_{3}=-0.65; \beta_{4}=-0.0001; \beta_{5}=1$ | |

**Missing data generating code in R**

library(foreign) # required to save data in .dta

library(descr)

library(dplyr)

## Import 2000 simulated datasets

datasets <- paste0("C:/completedata/dataset",1:2000,".dta")

data <- mclapply(datasets, read.dta)

MISSING10 <- function(ds){

set.seed(2000)

ds$totalcost <- sum(ds$cT1 + ds$cT2 + ds$cT3 + ds$cT4)

ds$mediantotalcost <- median(ds$totalcost)

ds$QALYs <- sum(ds$uT1 + ds$uT2 + ds$uT3 + ds$uT4)/4

ds$medianQALYs <- median(ds$QALYs)

ds$z1 <- ifelse(ds$trt == 1, 0.110*ds$age + 1*ds$gender + 0.65*ds$uT0 + 0.00027*ds$cT0 + 1*ds$trt, 1)

ds$z1 <- ifelse(ds$trt == 0, 0.110*ds$age - 1*ds$gender - 0.65*ds$uT0 - 0.00010*ds$cT0 + 1*ds$trt, ds$z1)

ds$pr1a = exp(-ds$z1)

ds$pr1b = 1 + ds$pr1a

ds$pr1c = 1 / ds$pr1b

ds$y1 = rbinom(600,1,ds$pr1c)

ds$uT1<-replace(ds$uT1, ds$y1==0,NA)

ds$cT1<-replace(ds$cT1, ds$y1==0,NA)

ds$M <- as.integer(complete.cases(ds$cT1))

descr(ds$M)

ds$uT2 <-replace(ds$uT2, ds$y1==0,NA)

ds$cT2 <-replace(ds$cT2, ds$y1==0,NA)

ds$z2 <- ifelse(ds$trt == 1, -0.1 + 0.100*ds$age + 1*ds$gender + 0.65*ds$uT0 + 0.00027*ds$cT0 + 1*ds$trt, 1)

ds$z2 <- ifelse(ds$trt == 0, -0.1 + 0.100*ds$age - 1*ds$gender - 0.65*ds$uT0 - 0.00027*ds$cT0 + 1*ds$trt, ds$z2)

ds$pr2a = exp(-ds$z2)

ds$pr2b = 1 + ds$pr2a

ds$pr2c = 1 / ds$pr2b

ds$y2 = rbinom(600,1,ds$pr2c)

ds$uT2<-replace(ds$uT2, ds$y2==0,NA)

ds$cT2<-replace(ds$cT2, ds$y2==0,NA)

ds$M2 <- as.integer(complete.cases(ds$cT2))

descr(ds$M2)

ds$uT3<-replace(ds$uT3, ds$y1==0,NA)

ds$cT3<-replace(ds$cT3, ds$y1==0,NA)

ds$uT3<-replace(ds$uT3, ds$y2==0,NA)

ds$cT3<-replace(ds$cT3, ds$y2==0,NA)

ds$z3 <- ifelse(ds$trt == 1, -0.2 + 0.90*ds$age + 1*ds$gender + 0.65*ds$uT0 + 0.00027*ds$cT0 + 1*ds$trt, 1)

ds$z3 <- ifelse(ds$trt == 0, -0.2 + 0.80*ds$age - 1*ds$gender - 0.65*ds$uT0 - 0.00010*ds$cT0 + 1*ds$trt, ds$z3)

ds$pr3a = exp(-ds$z3)

ds$pr3b = 1 + ds$pr3a

ds$pr3c = 1 / ds$pr3b

ds$y3 = rbinom(600,1,ds$pr3c)

ds$uT3<-replace(ds$uT3, ds$y3==0,NA)

ds$cT3<-replace(ds$cT3, ds$y3==0,NA)

ds$M3 <- as.integer(complete.cases(ds$cT3))

descr(ds$M3)

ds$uT4<-replace(ds$uT4, ds$y1==0,NA)

ds$cT4<-replace(ds$cT4, ds$y1==0,NA)

ds$uT4<-replace(ds$uT4, ds$y2==0,NA)

ds$cT4<-replace(ds$cT4, ds$y2==0,NA)

ds$uT4<-replace(ds$uT4, ds$y3==0,NA)

ds$cT4<-replace(ds$cT4, ds$y3==0,NA)

ds$z4 <- ifelse(ds$trt == 1, -1.9 + 0.100*ds$age + 1*ds$gender + 0.65*ds$uT0 + 0.00027*ds$cT0 + 1*ds$trt, 1)

ds$z4 <- ifelse(ds$trt == 0, -1.9 + 0.050*ds$age - 1*ds$gender - 0.65*ds$uT0 - 0.00010*ds$cT0 + 1*ds$trt, ds$z4)

ds$pr4a = exp(-ds$z4)

ds$pr4b = 1 + ds$pr4a

ds$pr4c = 1/ ds$pr4b

ds$y4 = rbinom(600,1,ds$pr4c)

ds$uT4<-replace(ds$uT4, ds$trt==1 & ds$y4==0 & ds$uT4 >= ds$mediantotalcost,NA)

ds$cT4<-replace(ds$cT4, ds$trt==0 & ds$y4==0 & ds$cT4 <= ds$medianQALYs,NA)

descr(ds$y4)

ds$M <- as.integer(complete.cases(ds))

descr(ds$M)

ds <- subset(ds,select =c("id","trt","age","gender","uT0","cT0","uT1","cT1","uT2","cT2","uT3","cT3","uT4","cT4"))

}

test <- lapply(data,MISSING10)

for (i in 1:2000) { write.dta(test[[i]], file = paste0("C:/MISSING10/dataset",i,".dta"))}

MISSING25 <- function(ds){

set.seed(2001)

ds$totalcost <- sum(ds$cT1 + ds$cT2 + ds$cT3 + ds$cT4)

ds$mediantotalcost <- median(ds$totalcost)

ds$QALYs <- sum(ds$uT1 + ds$uT2 + ds$uT3 + ds$uT4)/4

ds$medianQALYs <- median(ds$QALYs)

ds$z1 <- ifelse(ds$trt == 1, 0.110*ds$age + 1*ds$gender + 0.65*ds$uT0 + 0.00027*ds$cT0 + 1*ds$trt, 1)

ds$z1 <- ifelse(ds$trt == 0, 0.110*ds$age - 1*ds$gender - 0.65*ds$uT0 - 0.00010*ds$cT0 + 1*ds$trt, ds$z1)

ds$pr1a = exp(-ds$z1)

ds$pr1b = 1 + ds$pr1a

ds$pr1c = 1 / ds$pr1b

ds$y1 = rbinom(600,1,ds$pr1c)

ds$uT1<-replace(ds$uT1, ds$y1==0,NA)

ds$cT1<-replace(ds$cT1, ds$y1==0,NA)

ds$M <- as.integer(complete.cases(ds$cT1))

descr(ds$M)

ds$uT2 <-replace(ds$uT2, ds$y1==0,NA)

ds$cT2 <-replace(ds$cT2, ds$y1==0,NA)

ds$z2 <- ifelse(ds$trt == 1, -0.1 + 0.100*ds$age + 1*ds$gender + 0.65*ds$uT0 + 0.00027*ds$cT0 + 1*ds$trt, 1)

ds$z2 <- ifelse(ds$trt == 0, -0.1 + 0.100*ds$age - 1*ds$gender - 0.65*ds$uT0 - 0.00027*ds$cT0 + 1*ds$trt, ds$z2)

ds$pr2a = exp(-ds$z2)

ds$pr2b = 1 + ds$pr2a

ds$pr2c = 1 / ds$pr2b

ds$y2 = rbinom(600,1,ds$pr2c)

ds$uT2<-replace(ds$uT2, ds$y2==0,NA)

ds$cT2<-replace(ds$cT2, ds$y2==0,NA)

ds$M2 <- as.integer(complete.cases(ds$cT2))

descr(ds$M2)

ds$uT3<-replace(ds$uT3, ds$y1==0,NA)

ds$cT3<-replace(ds$cT3, ds$y1==0,NA)

ds$uT3<-replace(ds$uT3, ds$y2==0,NA)

ds$cT3<-replace(ds$cT3, ds$y2==0,NA)

ds$z3 <- ifelse(ds$trt == 1, -0.1 + 0.100*ds$age + 1*ds$gender + 0.65*ds$uT0 + 0.00027*ds$cT0 + 1*ds$trt, 1)

ds$z3 <- ifelse(ds$trt == 0, -0.1 + 0.033*ds$age - 1*ds$gender - 0.65*ds$uT0 - 0.00010*ds$cT0 + 1*ds$trt, ds$z3)

ds$pr3a = exp(-ds$z3)

ds$pr3b = 1 + ds$pr3a

ds$pr3c = 1 / ds$pr3b

ds$y3 = rbinom(600,1,ds$pr3c)

ds$uT3<-replace(ds$uT3, ds$y3==0,NA)

ds$cT3<-replace(ds$cT3, ds$y3==0,NA)

ds$M3 <- as.integer(complete.cases(ds$cT3))

descr(ds$M3)

ds$uT4<-replace(ds$uT4, ds$y1==0,NA)

ds$cT4<-replace(ds$cT4, ds$y1==0,NA)

ds$uT4<-replace(ds$uT4, ds$y2==0,NA)

ds$cT4<-replace(ds$cT4, ds$y2==0,NA)

ds$uT4<-replace(ds$uT4, ds$y3==0,NA)

ds$cT4<-replace(ds$cT4, ds$y3==0,NA)

ds$z4 <- ifelse(ds$trt == 1, + 0.300*ds$age + 1*ds$gender + 0.65*ds$uT0 + 0.00027*ds$cT0 + 1*ds$trt, 1)

ds$z4 <- ifelse(ds$trt == 0, - 0.300*ds$age - 1*ds$gender - 0.65*ds$uT0 - 0.00010*ds$cT0 + 1*ds$trt, ds$z4)

ds$pr4a = exp(-ds$z4)

ds$pr4b = 1 + ds$pr4a

ds$pr4c = 1/ ds$pr4b

ds$y4 = rbinom(600,1,ds$pr4c)

ds$uT4<-replace(ds$uT4, ds$trt==1 & ds$y4==0 & ds$uT4 >= ds$mediantotalcost,NA)

ds$cT4<-replace(ds$cT4, ds$trt==0 & ds$y4==0 & ds$cT4 <= ds$medianQALYs,NA)

descr(ds$y4)

ds$M4 <- as.integer(complete.cases(ds$cT4))

descr(ds$M4)

ds$M <- as.integer(complete.cases(ds))

descr(ds$M)

ds <- as.data.frame(ds)

ds <- subset(ds,select =c("id","trt","age","gender","uT0","cT0","uT1","cT1","uT2","cT2","uT3","cT3","uT4","cT4"))

}

test <- lapply(data,MISSING25)

for (i in 1:2000) {write.dta(test[[i]], file = paste0("C:/MISSING25/dataset",i,".dta"))}

MISSING50 <- function(ds){

set.seed(2012)

ds$totalcost <- sum(ds$cT1 + ds$cT2 + ds$cT3 + ds$cT4)

ds$mediantotalcost <- median(ds$totalcost)

ds$QALYs <- sum(ds$uT1 + ds$uT2 + ds$uT3 + ds$uT4)/4

ds$medianQALYs <- median(ds$QALYs)

ds$z1 <- ifelse(ds$trt == 1, -0.1 - 0.0715*ds$age + 1*ds$gender + 0.65*ds$uT0 + 0.00027*ds$cT0 + 1*ds$trt, 1)

ds$z1 <- ifelse(ds$trt == 0, -0.1 + 0.128*ds$age - 1*ds$gender - 0.65*ds$uT0 - 0.00010*ds$cT0 + 1*ds$trt, ds$z1)

ds$pr1a = exp(-ds$z1)

ds$pr1b = 1 + ds$pr1a

ds$pr1c = 1 / ds$pr1b

ds$y1 = rbinom(600,1,ds$pr1c)

ds$uT1<-replace(ds$uT1, ds$y1==0,NA)

ds$cT1<-replace(ds$cT1, ds$y1==0,NA)

ds$M <- as.integer(complete.cases(ds$cT1))

descr(ds$M)

ds$uT2 <-replace(ds$uT2, ds$y1==0,NA)

ds$cT2 <-replace(ds$cT2, ds$y1==0,NA)

ds$z2 <- ifelse(ds$trt == 1, -0.1 + 0.105*ds$age + 1*ds$gender + 0.65*ds$uT0 + 0.00027*ds$cT0 + 1*ds$trt, 1)

ds$z2 <- ifelse(ds$trt == 0, -0.1 + 0.128*ds$age - 1*ds$gender - 0.65*ds$uT0 - 0.00027*ds$cT0 + 1*ds$trt, ds$z2)

ds$pr2a = exp(-ds$z2)

ds$pr2b = 1 + ds$pr2a

ds$pr2c = 1 / ds$pr2b

ds$y2 = rbinom(600,1,ds$pr2c)

ds$uT2<-replace(ds$uT2, ds$y2==0,NA)

ds$cT2<-replace(ds$cT2, ds$y2==0,NA)

ds$M2 <- as.integer(complete.cases(ds$cT2))

descr(ds$M2)

ds$uT3<-replace(ds$uT3, ds$y1==0,NA)

ds$cT3<-replace(ds$cT3, ds$y1==0,NA)

ds$uT3<-replace(ds$uT3, ds$y2==0,NA)

ds$cT3<-replace(ds$cT3, ds$y2==0,NA)

ds$z3 <- ifelse(ds$trt == 1, -0.1 + 0.110*ds$age + 1*ds$gender + 0.65*ds$uT0 + 0.00027*ds$cT0 + 1*ds$trt, 1)

ds$z3 <- ifelse(ds$trt == 0, -0.1 + 0.130*ds$age - 1*ds$gender - 0.65*ds$uT0 - 0.00010*ds$cT0 + 1*ds$trt, ds$z3)

ds$pr3a = exp(-ds$z3)

ds$pr3b = 1 + ds$pr3a

ds$pr3c = 1 / ds$pr3b

ds$y3 = rbinom(600,1,ds$pr3c)

ds$uT3<-replace(ds$uT3, ds$y3==0,NA)

ds$cT3<-replace(ds$cT3, ds$y3==0,NA)

ds$M3 <- as.integer(complete.cases(ds$cT3))

descr(ds$M3)

ds$uT4<-replace(ds$uT4, ds$y1==0,NA)

ds$cT4<-replace(ds$cT4, ds$y1==0,NA)

ds$uT4<-replace(ds$uT4, ds$y2==0,NA)

ds$cT4<-replace(ds$cT4, ds$y2==0,NA)

ds$uT4<-replace(ds$uT4, ds$y3==0,NA)

ds$cT4<-replace(ds$cT4, ds$y3==0,NA)

ds$z4 <- ifelse(ds$trt == 1, + 0.300*ds$age + 1*ds$gender + 0.65*ds$uT0 + 0.00027*ds$cT0 + 1*ds$trt, 1)

ds$z4 <- ifelse(ds$trt == 0, - 0.300*ds$age - 1*ds$gender - 0.65*ds$uT0 - 0.00010*ds$cT0 + 1*ds$trt, ds$z4)

ds$pr4a = exp(-ds$z4)

ds$pr4b = 1 + ds$pr4a

ds$pr4c = 1/ ds$pr4b

ds$y4 = rbinom(600,1,ds$pr4c)

ds$uT4<-replace(ds$uT4, ds$trt==1 & ds$y4==0 & ds$uT4 >= ds$mediantotalcost,NA)

ds$cT4<-replace(ds$cT4, ds$trt==0 & ds$y4==0 & ds$cT4 <= ds$medianQALYs,NA)

descr(ds$y4)

ds$M4 <- as.integer(complete.cases(ds$cT4))

descr(ds$M4)

ds$M <- as.integer(complete.cases(ds))

descr(ds$M)

ds <- as.data.frame(ds)

ds <- subset(ds,select =c("id","trt","age","gender","uT0","cT0","uT1","cT1","uT2","cT2","uT3","cT3","uT4","cT4"))

}

test <- lapply(data,MISSING50)

for (i in 1:2000) {

write.dta(test[[i]], file = paste0("C:/MISSING50/dataset",i,".dta")}
