# Supplementary material for: The handling of missing data in trial-based economic evaluations: should data be multiply imputed prior to longitudinal linear mixed-model analyses?
Source: Eur J Health Econ. 2022 Sep 26;24(6):951–65. doi: 10.1007/s10198-022-01525-y (PMC10290620; doi:10.1007/s10198-022-01525-y)
Supplement: Supplementary file 4 — Supplementary file4 (DOCX 31 KB) [file 10198_2022_1525_MOESM4_ESM.docx]

**SUPPLEMENTARY MATERIAL 3**

**/* Stata code - LONGITUDINAL LINEAR MIXED-MODEL (LLM) */**

clear

set more off

cd "C:\MISSINGX"

local n = 2000

forvalues j = 1(1)`n' {

local y = `j'

use "dataset`y'", clear

//keep baseline values for costs and utilities in wide format

gen utility_b = uT0

gen cost_b = cT0

//reshape from wide to long creating a new variable - time - that indicates time period

quietly reshape long cT uT, i(id) j(time 0 1 2 3 4)

rename cT costs

rename uT utilities

//LLM FOR UTILITIES

mixed utilities i.trt##i.time i.time##c.utility_b i.time##c.age i.time##i.gender ||id :

mat betaCE = e(b) /* extract matric of betas */

mat vari = e(V) /* extract matrix of variances */

mat obs = e(N_g)

gen obs = obs[1,1] /* extract number of observations used in the model */

gen Za = 1.95996

//gen variables for utility difference at each time point adjusted for baseline

gen utility_diff1 = betaCE[1,14]

gen utility_diff2 = betaCE[1,15]

gen utility_diff3 = betaCE[1,16]

gen utility_diff4 = betaCE[1,17]

//calculate QALY difference

gen QALY_diff = (0.5*(utility_diff1+utility_diff1)*(3/12)) + (0.5*(utility_diff1+utility_diff2)*(3/12)) + (0.5*(utility_diff2+utility_diff3)*(3/12)) + (0.5*(utility_diff3+utility_diff4)*(3/12))

//gen variables for variance utility difference at each time point

gen varu1 = vari[14,14]

gen varu2 = vari[15,15]

gen varu3 = vari[16,16]

gen varu4 = vari[17,17]

//calculate variance of QALY difference

gen QALY_var = ((0.25*(varu1+varu1)) + (0.25*(varu1+varu2)) + (0.25*(varu2+varu3)) + (0.25*(varu3+varu4)))/4

//calculate SE of QALY difference

gen SE_QALY_diff = sqrt(QALY_var)

//estimate CI around QALY difference

gen LL_QALY = QALY_diff - Za*SE_QALY_diff

gen UL_QALY = QALY_diff + Za*SE_QALY_diff

//LLM FOR COSTS

mixed costs i.trt##i.time i.time##c.cost_b i.time##c.age i.time##i.gender ||id :

mat betaCEc = e(b) /* extract matrix of regression coefficients */

mat varic = e(V) /* extract matrix of variances */

mat obsc = e(N_g)

gen obsc = obsc[1,1] /* extract number of observations used in the model */

//gen variables for cost difference at each time point adjusted for baseline costs

gen cost_diff1 = betaCEc[1,14]

gen cost_diff2 = betaCEc[1,15]

gen cost_diff3 = betaCEc[1,16]

gen cost_diff4 = betaCEc[1,17]

//calculate marginal cost difference

gen cost_diff = cost_diff1 + cost_diff2 + cost_diff3 + cost_diff4

//gen variables for variance of cost difference at each time point

gen varc1 = varic[14,14]

gen varc2 = varic[15,15]

gen varc3 = varic[16,16]

gen varc4 = varic[17,17]

//calculate SE for marginal cost difference

gen SEc1 = sqrt(varc1)

gen SEc2 = sqrt(varc2)

gen SEc3 = sqrt(varc3)

gen SEc4 = sqrt(varc4)

gen SE_cost_diff = SEc1 + SEc2 + SEc3 + SEc4

//estimate CI around marginal cost difference

gen LL_costs = (cost_diff - Za*SE_cost_diff)

gen UL_costs = (cost_diff + Za*SE_cost_diff)

//calculate covariance costs and QALY

cor costs utilities if trt == 0

mat cor = r(rho)

gen cor_control = cor[1,1]

cor costs utilities if trt == 1

mat cor = r(rho)

gen cor_intervention = cor[1,1]

tabstat costs, stats(sd) save by(trt)

mat sd = r(Stat1)

gen Costs_SD_control = sd[1,1]

tabstat costs, stats(sd) save by(trt)

mat sd = r(Stat2)

gen Costs_SD_intervention = sd[1,1]

tabstat utilities, stats(sd) save by(trt)

mat sd = r(Stat1)

gen QALY_SD_control = sd[1,1]

tabstat utilities, stats(sd) save by(trt)

mat sd = r(Stat2)

gen QALY_SD_intervention = sd[1,1]

gen QALY_control_se = QALY_SD_control/sqrt(obs)

gen QALY_int_se = QALY_SD_intervention/sqrt(obs)

gen Costs_control_se = Costs_SD_control/sqrt(obs)

gen Costs_int_se = Costs_SD_intervention/sqrt(obs)

gen se_QALY = sqrt(QALY_int_se^2+QALY_control_se^2)

gen se_Costs = sqrt(Costs_int_se^2+Costs_control_se^2)

gen cor_cost_effect_diffs = ((cor_intervention*QALY_int_se*Costs_int_se)+(cor_control*QALY_control_se*Costs_control_se))/(se_QALY*se_Costs)

gen cov = cor_cost_effect_diffs*SE_QALY_diff*SE_cost_diff

save "LLM\postboots`y'", replace

}

**/* MULTIPLE IMPUTATION + LONGITUDINAL LINEAR MIXED-MODEL (MI-LLM) */**

clear

set more off

cd "C:\MISSINGX"

local n = 2000

forvalues j = 1(1)`n' {

local y = `j'

use "dataset`y'", clear

// MULTIPLE IMPUTATION MODEL

mi set flong

mi register regular age gender trt uT0 cT0

mi register imputed cT1 cT2 cT3 cT4 uT1 uT2 uT3 uT4

quietly mi impute chained (pmm, knn(5)) cT1 cT2 cT3 cT4 uT1 uT2 uT3 uT4 = age gender uT0 cT0, by(trt) replace add(10) rseed(`k')

save "C:\MISSINGX\MI-LLM\dataset`y'_imp", replace

}

clear

set more off

cd "C:\MISSING\MI-LLM"

local n = 2000

forvalues j = 1(1)`n' {

local y = `j'

use "dataset`y'_imp", clear

//keep baseline values for costs and utilities in wide format

gen utility_b = uT0

gen cost_b = cT0

//reshape from wide to long creating a new variable - time - that indicates time period

rename (cT0 cT1 cT2 cT3 cT4) (cost0 cost1 cost2 cost3 cost4)

rename (uT0 uT1 uT2 uT3 uT4) (utility0 utility1 utility2 utility3 utility4)

quietly mi reshape long cost utility, i(id) j(time 0 1 2 3 4)

//LLM FOR UTILITIES performed in each on of the imputed datasets

quietly mi estimate: mixed utility i.trt##i.time i.time##c.utility_b i.time##c.age i.time##i.gender ||id :

mat betaCE = e(b_mi) /* extract matric of betas */

mat vari = e(V_mi) /* extract matrix of variances */

mat obs = e(N_g_mi) /* extract number of observations used in the model */

gen obs = obs[1,1]

gen loss_eff = e(fmi_max_mi)/e(M_mi)

gen Za = 1.95996

//gen variables for utility difference at each time point adjusted for baseline */

gen utility_diff1 = betaCE[1,14]

gen utility_diff2 = betaCE[1,15]

gen utility_diff3 = betaCE[1,16]

gen utility_diff4 = betaCE[1,17]

//calculate QALY difference

gen QALY_diff = (0.5*(utility_diff1+utility_diff1)*(3/12)) + (0.5*(utility_diff1+utility_diff2)*(3/12)) + (0.5*(utility_diff2+utility_diff3)*(3/12)) + (0.5*(utility_diff3+utility_diff4)*(3/12))

//gen variables for variance utility difference at each time point

gen varu1 = vari[14,14]

gen varu2 = vari[15,15]

gen varu3 = vari[16,16]

gen varu4 = vari[17,17]

//calculate variance of QALY difference

gen QALY_var = ((0.25*(varu1+varu1)) + (0.25*(varu1+varu2)) + (0.25*(varu2+varu3)) + (0.25*(varu3+varu4)))/4

//calculate SE of QALY difference

gen SE_QALY_diff = sqrt(QALY_var)

//estimate CI around QALY difference

gen LL_QALY = QALY_diff - Za*SE_QALY_diff

gen UL_QALY = QALY_diff + Za*SE_QALY_diff

//LLM FOR COSTS performed in each on of the imputed datasets

quietly mi estimate: mixed cost i.trt##i.time i.time##c.cost_b i.time##c.age i.time##i.gender ||id :

mat betaCEc = e(b_mi) /* extract matric of betas */

mat varic = e(V_mi) /* extract matrix of variances */

mat obsc = e(N_g_mi) /* extract number of observations used in the model */

gen obsc = obsc[1,1]

gen loss_effc = e(fmi_max_mi)/e(M_mi)

//gen variables for cost difference at each time point adjusted for baseline costs

gen cost_diff1 = betaCEc[1,14]

gen cost_diff2 = betaCEc[1,15]

gen cost_diff3 = betaCEc[1,16]

gen cost_diff4 = betaCEc[1,17]

//calculate marginal cost difference

gen cost_diff = cost_diff1 + cost_diff2 + cost_diff3 + cost_diff4

//gen variables for variance of cost difference at each time point

gen varc1 = varic[14,14]

gen varc2 = varic[15,15]

gen varc3 = varic[16,16]

gen varc4 = varic[17,17]

//calculate SE for marginal cost difference

gen SEc1 = sqrt(varc1)

gen SEc2 = sqrt(varc2)

gen SEc3 = sqrt(varc3)

gen SEc4 = sqrt(varc4)

gen SE_cost_diff = SEc1 + SEc2 + SEc3 + SEc4

//estimate CI around marginal cost difference

gen LL_costs = (cost_diff - Za*SE_cost_diff)

gen UL_costs = (cost_diff + Za*SE_cost_diff)

//calculate covariance costs and QALY

cor cost utility if trt == 0

mat cor = r(rho)

gen cor_control = cor[1,1]

cor cost utility if trt == 1

mat cor = r(rho)

gen cor_intervention = cor[1,1]

tabstat cost, stats(sd) save by(trt)

mat sd = r(Stat1)

gen Costs_SD_control = sd[1,1]

tabstat cost, stats(sd) save by(trt)

mat sd = r(Stat2)

gen Costs_SD_intervention = sd[1,1]

tabstat utility, stats(sd) save by(trt)

mat sd = r(Stat1)

gen QALY_SD_control = sd[1,1]

tabstat utility, stats(sd) save by(trt)

mat sd = r(Stat2)

gen QALY_SD_intervention = sd[1,1]

gen QALY_control_se = QALY_SD_control/sqrt(obs)

gen QALY_int_se = QALY_SD_intervention/sqrt(obs)

gen Costs_control_se = Costs_SD_control/sqrt(obs)

gen Costs_int_se = Costs_SD_intervention/sqrt(obs)

gen se_QALY = sqrt(QALY_int_se^2+QALY_control_se^2)

gen se_Costs = sqrt(Costs_int_se^2+Costs_control_se^2)

gen cor_cost_effect_diffs = ((cor_intervention*QALY_int_se*Costs_int_se)+(cor_control*QALY_control_se*Costs_control_se))/(se_QALY*se_Costs)

gen cov = cor_cost_effect_diffs*SE_QALY_diff*SE_cost_diff

save "C:\MISSINGX\MI-LLM\postboots`y'", replace

}

/* **MEAN IMPUTATION + LONGITUDINAL LINEAR MIXED-MODEL (M-LLM)** */

clear

set more off

cd "C:\MISSINGX"

local n = 2000

forvalues j = 1(1)`n' {

local y = `j'

use "dataset`y'", clear

// MEAN IMPUTATION BY TREATMENT GROUP

bysort trt: egen mean_uT1 = mean(uT1)

bysort trt: egen mean_uT2 = mean(uT2)

bysort trt: egen mean_uT3 = mean(uT3)

bysort trt: egen mean_uT4 = mean(uT4)

replace uT1 = mean_uT1 if missing(uT1)

replace uT2 = mean_uT2 if missing(uT2)

replace uT3 = mean_uT3 if missing(uT3)

replace uT4 = mean_uT4 if missing(uT4)

bysort trt: egen mean_cT1 = mean(cT1)

bysort trt: egen mean_cT2 = mean(cT2)

bysort trt: egen mean_cT3 = mean(cT3)

bysort trt: egen mean_cT4 = mean(cT4)

replace cT1 = mean_cT1 if missing(cT1)

replace cT2 = mean_cT2 if missing(cT2)

replace cT3 = mean_cT3 if missing(cT3)

replace cT4 = mean_cT4 if missing(cT4)

//keep baseline values for costs and utilities in wide format

gen utility_b = uT0

gen cost_b = cT0

//reshape from wide to long creating a new variable - time - that indicates time period

quietly reshape long cT uT, i(id) j(time 0 1 2 3 4)

rename cT costs

rename uT utilities

//LLM FOR UTILITIES

mixed utilities i.trt##i.time i.time##c.utility_b i.time##c.age i.time##i.gender ||id :

mat betaCE = e(b) /* extract matric of betas */

mat vari = e(V) /* extract matrix of variances */

mat obs = e(N_g)

gen obs = obs[1,1] /* extract number of observations used in the model */

gen Za = 1.95996

//gen variables for utility difference at each time point adjusted for baseline

gen utility_diff1 = betaCE[1,14]

gen utility_diff2 = betaCE[1,15]

gen utility_diff3 = betaCE[1,16]

gen utility_diff4 = betaCE[1,17]

//calculate QALY difference

gen QALY_diff = (0.5*(utility_diff1+utility_diff1)*(3/12)) + (0.5*(utility_diff1+utility_diff2)*(3/12)) + (0.5*(utility_diff2+utility_diff3)*(3/12)) + (0.5*(utility_diff3+utility_diff4)*(3/12))

//gen variables for variance utility difference at each time point

gen varu1 = vari[14,14]

gen varu2 = vari[15,15]

gen varu3 = vari[16,16]

gen varu4 = vari[17,17]

//calculate variance of QALY difference

gen QALY_var = ((0.25*(varu1+varu1)) + (0.25*(varu1+varu2)) + (0.25*(varu2+varu3)) + (0.25*(varu3+varu4)))/4

//calculate SE of QALY difference

gen SE_QALY_diff = sqrt(QALY_var)

//estimate CI around QALY difference

gen LL_QALY = QALY_diff - Za*SE_QALY_diff

gen UL_QALY = QALY_diff + Za*SE_QALY_diff

//LLM FOR COSTS

mixed costs i.trt##i.time i.time##c.cost_b i.time##c.age i.time##i.gender ||id :

mat betaCEc = e(b) /* extract matrix of regression coefficients */

mat varic = e(V) /* extract matrix of variances */

mat obsc = e(N_g)

gen obsc = obsc[1,1] /* extract number of observations used in the model */

//gen variables for cost difference at each time point adjusted for baseline costs

gen cost_diff1 = betaCEc[1,14]

gen cost_diff2 = betaCEc[1,15]

gen cost_diff3 = betaCEc[1,16]

gen cost_diff4 = betaCEc[1,17]

//calculate marginal cost difference

gen cost_diff = cost_diff1 + cost_diff2 + cost_diff3 + cost_diff4

//gen variables for variance of cost difference at each time point

gen varc1 = varic[14,14]

gen varc2 = varic[15,15]

gen varc3 = varic[16,16]

gen varc4 = varic[17,17]

//calculate SE for marginal cost difference

gen SEc1 = sqrt(varc1)

gen SEc2 = sqrt(varc2)

gen SEc3 = sqrt(varc3)

gen SEc4 = sqrt(varc4)

gen SE_cost_diff = SEc1 + SEc2 + SEc3 + SEc4

//estimate CI around marginal cost difference

gen LL_costs = (cost_diff - Za*SE_cost_diff)

gen UL_costs = (cost_diff + Za*SE_cost_diff)

//calculate covariance costs and QALY

cor costs utilities if trt == 0

mat cor = r(rho)

gen cor_control = cor[1,1]

cor costs utilities if trt == 1

mat cor = r(rho)

gen cor_intervention = cor[1,1]

tabstat costs, stats(sd) save by(trt)

mat sd = r(Stat1)

gen Costs_SD_control = sd[1,1]

tabstat costs, stats(sd) save by(trt)

mat sd = r(Stat2)

gen Costs_SD_intervention = sd[1,1]

tabstat utilities, stats(sd) save by(trt)

mat sd = r(Stat1)

gen QALY_SD_control = sd[1,1]

tabstat utilities, stats(sd) save by(trt)

mat sd = r(Stat2)

gen QALY_SD_intervention = sd[1,1]

gen QALY_control_se = QALY_SD_control/sqrt(obs)

gen QALY_int_se = QALY_SD_intervention/sqrt(obs)

gen Costs_control_se = Costs_SD_control/sqrt(obs)

gen Costs_int_se = Costs_SD_intervention/sqrt(obs)

gen se_QALY = sqrt(QALY_int_se^2+QALY_control_se^2)

gen se_Costs = sqrt(Costs_int_se^2+Costs_control_se^2)

gen cor_cost_effect_diffs = ((cor_intervention*QALY_int_se*Costs_int_se)+(cor_control*QALY_control_se*Costs_control_se))/(se_QALY*se_Costs)

gen cov = cor_cost_effect_diffs*SE_QALY_diff*SE_cost_diff

save "M-LLM\postboots`y'", replace

}

**/*SEEMINGLY UNRELATED REGRESSION COMPLETE CASE ANALYSIS (SUR)*/**

clear

set more off

cd "C:\MISSINGX"

local n = 2000

forvalues j = 1(1)`n' {

local y = `j'

use "dataset`y'", clear

gen QALY = (0.5*(uT1+uT1)*(3/12)) + (0.5*(uT1+uT2)*(3/12)) + (0.5*(uT2+uT3)*(3/12)) + (0.5*(uT3+uT4)*(3/12))

gen Tcosts = cT1 + cT2 + cT3 + cT4

quietly sureg (Tcosts=trt cT0 age gender) (QALY=trt uT0 age gender)

mat betaCE= e(b) /* extracT the matrix of regression coefficients */

mat vari = e(V) /* extracT matrix of variances */

gen obs = e(N) /* extracT number of observations used in the model */

gen cost_diff = betaCE[1,1] /* generate cost difference */

gen QALY_diff = betaCE[1,6] /* generate QALY difference */

gen cost_var = vari[1,1] /* generate variance of cost difference */

gen QALY_var = vari[6,6] /* generate variance of QALY difference */

gen cov = vari[1,6] /* generate covariance cost and QALY difference */

gen SE_cost_diff = sqrt(cost_var)

gen SE_QALY_diff = sqrt(QALY_var)

gen Za = 1.95996

gen LL_costs = cost_diff - Za*SE_cost_diff /* lower CI cost difference */

gen UL_costs = cost_diff + Za*SE_cost_diff /* upper CI cost difference */

gen LL_QALY = QALY_diff - Za*SE_QALY_diff /* lower CI QALY difference */

gen UL_QALY = QALY_diff + Za*SE_QALY_diff /* upper CI QALY difference */

save "SUREG\postboots`y'", replace

}

**/* MULTIPLE IMPUTATION + SEEMINGLY UNRELATE REGRESSION (MI-SUR) */**

clear

set more off

cd "C:\MISSINGX"

local n = 2000

forvalues k=1(1)`n' {

local y = `k'

use "dataset`y'", clear

// MULTIPLE IMPUTATION MODEL

mi set flong

mi register regular age gender trt uT0 cT0

mi register imputed cT1 cT2 cT3 cT4 uT1 uT2 uT3 uT4

quietly mi impute chained (pmm, knn(5)) cT1 cT2 cT3 cT4 uT1 uT2 uT3 uT4 = age gender uT0 cT0, by(trt) replace add(10) rseed(`k')

save "C:\MISSINGX\MI-LLM\dataset`y'_imp", replace

save "C:\MISSINGX\MI-SUREG\dataset`y'_imp", replace

gen QALY = (0.5*(uT1+uT1)*(3/12)) + (0.5*(uT1+uT2)*(3/12)) + (0.5*(uT2+uT3)*(3/12)) + (0.5*(uT3+uT4)*(3/12))

gen Tcosts = cT1 + cT2 + cT3 + cT4

quietly mi estimate, cmdok: sureg (Tcosts=trt cT0 age gender) (QALY=trt uT0 age gender)

gen obs = e(N_mi)

gen loss_eff = e(fmi_max_mi)/e(M_mi)

mat betaCE= e(b_mi) /* extract the matrix of regression coefficients */

mat vari = e(V_mi) /* extract matrix of variances */

gen cost_diff = betaCE[1,1] /* generate cost difference */

gen QALY_diff = betaCE[1,6] /* generate QALY difference */

gen cost_var = vari[1,1] /* generate variance of cost difference */

gen QALY_var = vari[6,6] /* generate variance of QALY difference */

gen cov = vari[1,6] /* generate covariance cost and QALY difference */

gen SE_cost_diff = sqrt(cost_var)

gen SE_QALY_diff = sqrt(QALY_var)

gen Za = 1.95996

gen LL_costs = cost_diff - Za*SE_cost_diff /* lower CI cost difference */

gen UL_costs = cost_diff + Za*SE_cost_diff /* upper CI cost difference */

gen LL_QALY = QALY_diff - Za*SE_QALY_diff /* lower CI QALY difference */

gen UL_QALY = QALY_diff + Za*SE_QALY_diff /* upper CI QALY difference */

save "C:\MISSINGX\MI-SUREG\postboots`y'", replace

}

**/* MEAN IMPUTATION + SEEMENGLY UNRELATED REGRESSION (M-SUR) */**

clear

set more off

cd "C:\MISSINGX"

local n = 2000

forvalues j = 1(1)`n' {

local y = `j'

use "dataset`y'", clear

// MEAN IMPUTATION BY TREATMENT GROUP

bysort trt: egen mean_uT1 = mean(uT1)

bysort trt: egen mean_uT2 = mean(uT2)

bysort trt: egen mean_uT3 = mean(uT3)

bysort trt: egen mean_uT4 = mean(uT4)

replace uT1 = mean_uT1 if missing(uT1)

replace uT2 = mean_uT2 if missing(uT2)

replace uT3 = mean_uT3 if missing(uT3)

replace uT4 = mean_uT4 if missing(uT4)

bysort trt: egen mean_cT1 = mean(cT1)

bysort trt: egen mean_cT2 = mean(cT2)

bysort trt: egen mean_cT3 = mean(cT3)

bysort trt: egen mean_cT4 = mean(cT4)

replace cT1 = mean_cT1 if missing(cT1)

replace cT2 = mean_cT2 if missing(cT2)

replace cT3 = mean_cT3 if missing(cT3)

replace cT4 = mean_cT4 if missing(cT4)

gen QALY = (0.5*(uT1+uT1)*(3/12)) + (0.5*(uT1+uT2)*(3/12)) + (0.5*(uT2+uT3)*(3/12)) + (0.5*(uT3+uT4)*(3/12))

gen Tcosts = cT1 + cT2 + cT3 + cT4

quietly sureg (Tcosts=trt cT0 age gender) (QALY=trt uT0 age gender)

mat betaCE= e(b) /* extract the matrix of regression coefficients */

mat vari = e(V) /* extract matrix of variances */

gen obs = e(N) /* extract number of observations used in the model */

gen cost_diff = betaCE[1,1] /* generate cost difference */

gen QALY_diff = betaCE[1,6] /* generate QALY difference */

gen cost_var = vari[1,1] /* generate variance of cost difference */

gen QALY_var = vari[6,6] /* generate variance of QALY difference */

gen cov = vari[1,6] /* generate covariance cost and QALY difference */

gen SE_cost_diff = sqrt(cost_var)

gen SE_QALY_diff = sqrt(QALY_var)

gen Za = 1.95996

gen LL_costs = cost_diff - Za*SE_cost_diff /* lower CI cost difference */

gen UL_costs = cost_diff + Za*SE_cost_diff /* upper CI cost difference */

gen LL_QALY = QALY_diff - Za*SE_QALY_diff /* lower CI QALY difference */

gen UL_QALY = QALY_diff + Za*SE_QALY_diff /* upper CI QALY difference */

save "M-SUREG\postboots`y'", replace

}
