# Supplementary material for: The handling of missing data in trial-based economic evaluations: should data be multiply imputed prior to longitudinal linear mixed-model analyses?
Source: Eur J Health Econ. 2022 Sep 26;24(6):951–65. doi: 10.1007/s10198-022-01525-y (PMC10290620; doi:10.1007/s10198-022-01525-y)
Supplement: Supplementary file 5 — Supplementary file5 (DOCX 168 KB) [file 10198_2022_1525_MOESM5_ESM.docx]

**SUPPLEMENTARY MATERIAL 4**

**Empirical dataset 1**

Data from two pragmatic randomized controlled trials were used in addition to the simulated data. In the first trial (empirical dataset 1), the cost-effectiveness of early rehabilitation after lumbar disc surgery was compared to no referral[18]. For the current study, utility values collected at baseline, 12, and 26 weeks and costs collected at 6, 12, and 26 weeks were used. For all scenarios, mean imputation was used to impute missing values at baseline[5]. Of the 169 participants used in our study, 13% (n=22) had missing cost and/or utility data at one or more follow-up time points. Stepwise backwards regression models with p<0.05, were used to identify baseline variables that were predictive of the missingness of data and/or the cost-effectiveness outcomes. The identified variables were added to the imputation model as auxiliary variables (i.e. age, level of education, utility values, Oswestry Disability Index [ODI], pain intensity, Örebro Musculoskeletal Pain Screening Questionnaire [OMPSQ], and the credibility and expectancy surgery [CEQ])[18]. Missing cost and utility data were imputed using Multivariate Imputation by Chained Equations (MICE; FCS-standard)[33] with PMM, stratified by treatment group[34]. Ten datasets were imputed to guarantee a loss of efficiency <0.05. SUR and LLM were then fitted to the imputed data. The LLM analysis model included all auxiliary variables as, in doing so, it should lead to similar results when compared to MI-LL[5]. The other LMM models (i.e., M-LLM and MI-LLM) and SUR models (i.e., SUR-CCA, M-SUR, and MI-SUR) did not include auxiliary variables, and only were corrected for confounders (i.e. baseline utility values, ODI, OMPSQ, and CEQ).

**Longitudinal Linear Mixed-model analysis (LLM)** – Two separate LLMs were performed, including one for costs and one for utility values:

${Costs}_{ij}= \beta_{1c}{time}_{j}+{\beta_{2c}{trt}_{i}+\beta}_{3c}{time}_{j}{trt}_{i}+ \beta_{4c}{uT0}_{i}+{\beta_{5c}{NRS}_{i}+\beta}_{6c}{ODI}_{i}+\beta_{7c}{CEQ}_{i}+ \omega_{ci}+ \varepsilon_{cij}$,

${Utility}_{ij}= \beta_{1u}{time}_{j}+{\beta_{2u}{trt}_{i}+\beta}_{3u}{time}_{j}{trt}_{i}+ \beta_{4u}{uT0}_{i}+ \beta_{5u}{age}_{i}+{\beta_{6u}{educ}_{i}+\beta}_{7u}{OMPSQ}_{i}+ \beta_{8u}{CEQ}_{i}+ \omega_{ui}+ \varepsilon_{uij}$,

${\omega_{i} \sim Normal(0, \sigma_{\omega}^{2}), \varepsilon}_{ij} \sim Normal(0, \sigma_{\varepsilon}^{2}$)

where ${Costs}_{ij}$ and ${Utility}_{ij}$ represent the cost and utility values of subject *i* (*i* = 1, …, N=169) at time point *j* (*j* = 1, …, 3). The model parameters include the intercept $\beta_{1}$ and the coefficients ${\beta_{2, \ldots,}\beta}_{n}$ of covariates including ${time}_{j}$ as an interaction term for the treatment effect. $\omega_{ci}$ and $\omega_{ui}$represent the random intercepts and $\varepsilon_{ij}$ and $\varepsilon_{uij}$represent the error term for a patient *i* at each time point *j* for *Costs* and *Utility*, respectively. Both $\omega_{i}$ and $\varepsilon_{ij}$follow a normal distribution.

**Mean Imputation combined with LLM (M-LLM)** – In this strategy, missing cost and utility values were replaced by the mean values from the available cases at each time point (i.e., unconditional mean imputation)[5]. Subsequently, two separate LLMs were fitted but not including the variables associated with missingness:

${Costs}_{ij}= \beta_{1c}{time}_{j}+{\beta_{2c}{trt}_{i}+\beta}_{3c}{time}_{j}{trt}_{i}+ \beta_{4c}{uT0}_{i}+\beta_{5c}{ODI}_{i}+\beta_{6c}{CEQ}_{i}+ \omega_{ci}+ \varepsilon_{cij}$,

${Utility}_{ij}= \beta_{1u}{time}_{j}+{\beta_{2u}{trt}_{i}+\beta}_{3u}{time}_{j}{trt}_{i}+ \beta_{4u}{uT0}_{i} {+\beta}_{5u}{OMPSQ}_{i}+ \beta_{6u}{CEQ}_{i}+ \omega_{ui}+ \varepsilon_{uij}$,

${\omega_{i} \sim Normal(0, \sigma_{\omega}^{2}), \varepsilon}_{ij} \sim Normal(0, \sigma_{\varepsilon}^{2}$)

**Multiple Imputation combined with LLM (MI-LLM)** – In this strategy, missing cost and utility values were first imputed using Multivariate Imputation by Chained Equations (MICE; FCS-standard)[32] with Predictive Mean Matching (PMM) by treatment group as outlined above[33]. Subsequently, two separate LLMs were fitted but not including the variables associated with missingness as show in the M-LLM strategy.

**Seemingly Unrelated Regressions - Complete Case Analysis (SUR-CCA)** – In this strategy, all subjects with missing values were deleted from the datasets (i.e. a complete case analysis). Then, total costs and QALYs were calculated by adding costs at each time point and using the area under the curve method, respectively[23]. Total cost and QALY differences between treatment groups were estimated using seemingly unrelated regressions (SUR). With SUR two regression equations are modelled simultaneously (i.e., one for total costs and one for QALY), while correcting for their possible correlation through correlated error terms[16, 34]:

$${Costs}_{i}= \beta_{0c}+ \beta_{1c}{trt}_{i}+ \beta_{2c}{ODI}_{i}+ \beta_{3c}{CEQ}_{i} +\varepsilon_{ci}$$

$${QALYs}_{i}= \beta_{0q}+ \beta_{1q}{trt}_{i}+ \beta_{2q}{uT0}_{i}+ \beta_{3q}{OMPSQ}_{i}+ \beta_{4q}{CEQ}_{i}+ \varepsilon_{qi}$$

$\left( \begin{aligned} \varepsilon_{ci} \\ \varepsilon_{qi} \end{aligned} \right)\sim Normal\left( \left( \begin{aligned} 0 \\ 0 \end{aligned} \right), \left( \begin{matrix} \sigma_{c}^{2} & \sigma_{cq} \\ \sigma_{cq} & \sigma_{q}^{2} \end{matrix} \right) \right)$

**Mean Imputation combined with SUR (M-SUR)** – In this strategy, missing cost and utility data were replaced by the mean values from the available cases at each time point (i.e., unconditional mean imputation)[5]. Subsequently, total costs and QALYs were calculated, and SUR analyses were performed as outlined under SUR.

**Multiple Imputation combined with SUR (MI-SUR)** – In this strategy, missing cost and utility data were first imputed using Multivariate Imputation by Chained Equations (MICE) as outlined above. Then, total costs and QALYs were calculated and a SUR was fitted per imputed dataset as outlined under SUR, after which pooled estimates were obtained using Rubin’s rules[32]. The SUR model as fitted as shown by SUR equations above.

**Empirical dataset 2**

In the second trial (empirical dataset 2), the cost-effectiveness of an interpersonal psychotherapy for older adults with major depression was compared to care as usual (i.e., control). For this study, utility values collected at baseline, 6, and 12 months and costs collected at 2, 6, and 12 months were used[19]. Mean imputation was used to impute missing values at baseline[5]. Of the 143 participants, 68% (n=98) of cost and utility data were missing at one or more follow-up time points. Stepwise backwards regression models with p<0.05, were used to identify baseline variables that were predictive of the missingness of data and/or the cost-effectiveness outcomes. The identified variables were added to the imputation model as auxiliary variables (i.e., age, activity daily living [ADL], utility values, alcohol-induced disorder, and mental health problems utility values, marital status, and household composition) [19]. Missing cost and utility data were imputed using Multivariate Imputation by Chained Equations (MICE; FCS-standard)[33] with PMM by treatment group[34]. Twenty datasets were imputed to guarantee a loss of efficiency <0.05. SUR and LLM were then fitted to the imputed data. The LLM analysis model included all auxiliary variables[5]. The other LMM models (i.e., M-LLM and MI-LLM) and SUR models (i.e., SUR-CCA, M-SUR, and MI-SUR) did not include auxiliary variables and only were corrected for confounders (i.e., baseline utility values, marital status, and household composition).

**Longitudinal Linear Mixed-model analysis (LLM)** – Two separate LLMs were performed, including one for costs and one for utility values:

${Costs}_{ij}= \beta_{1c}{time}_{j}+{\beta_{2c}{trt}_{i}+\beta}_{3c}{time}_{j}{trt}_{i}+ \beta_{4c}{uT0}_{i}+ \beta_{5c}{ALC}_{i}+{\beta_{6c}{MS}_{i}+\beta}_{7c}SF{36mh}_{i}+ \omega_{ci}+ \varepsilon_{cij}$,

${Utility}_{ij}= \beta_{1u}{time}_{j}+{\beta_{2u}{trt}_{i}+\beta}_{3u}{time}_{j}{trt}_{i}+ \beta_{4u}{uT0}_{i}+ \beta_{5u}{age}_{i}+{\beta_{6u}{ADL}_{i}+\beta}_{7u}{HC}_{i}+ \beta_{8u}{MS}_{i}+ \omega_{ui}+ \varepsilon_{uij}$,

${\omega_{i} \sim Normal(0, \sigma_{\omega}^{2}), \varepsilon}_{ij} \sim Normal(0, \sigma_{\varepsilon}^{2}$)

where ${Costs}_{ij}$ and ${Utility}_{ij}$ represent the cost and utility values of subject *i* (*i* = 1, …, N=143) at time point *j* (*j* = 1, …, 3). The model parameters include the intercept $\beta_{1}$ and the coefficients ${\beta_{2, \ldots,}\beta}_{n}$ of covariates including ${time}_{j}$ as an interaction term for the treatment effect. $\omega_{ci}$ and . $\omega_{ui}$ represent the random intercepts and $\varepsilon_{cij}$ and $\varepsilon_{uij}$ represent the error terms for a patient *i* at each time point *j* for *Costs* and *Utility*, respectively. Both $\omega_{i}$ and $\varepsilon_{ij}$follow a normal distribution.

**Mean Imputation combined with LLM (M-LLM)** – In this strategy, missing cost and utility values were replaced by the mean values from the available cases at each time point (i.e., unconditional mean imputation)[5]. Subsequently, two separate LLMs were fitted but not including the variables associated with missingness:

${Costs}_{ij}= \beta_{1c}{time}_{j}+{\beta_{2c}{trt}_{i}+\beta}_{3c}{time}_{j}{trt}_{i}+ \beta_{4c}{uT0}_{i}+\beta_{5c}{MS}_{i}+ \omega_{ci}+ \varepsilon_{cij}$,

${Utility}_{ij}= \beta_{1u}{time}_{j}+{\beta_{2u}{trt}_{i}+\beta}_{3u}{time}_{j}{trt}_{i}+ \beta_{4u}{uT0}_{i} {+\beta}_{5u}{MS}_{i}+ \beta_{6u}{HC}_{i}+ \omega_{ui}+ \varepsilon_{uij}$,

${\omega_{i} \sim Normal(0, \sigma_{\omega}^{2}), \varepsilon}_{ij} \sim Normal(0, \sigma_{\varepsilon}^{2}$)

**Multiple Imputation combined with LLM (MI-LLM)** – In this strategy, missing cost and utility values were first imputed using Multivariate Imputation by Chained Equations (MICE; FCS-standard)[32] with Predictive Mean Matching (PMM) by treatment group as outlined above[33]. Subsequently, two separate LLMs were fitted but not including the variables associated with missingness as show in the M-LLM strategy.

**Seemingly Unrelated Regressions - Complete Case Analysis (SUR-CCA)** – In this strategy, all subjects with missing values were deleted from the datasets (i.e. a complete case analysis). Then, total costs and QALYs were calculated by adding costs at each time point and using the area under the curve method, respectively[23]. Total cost and QALY differences between treatment groups were estimated using seemingly unrelated regressions (SUR). With SUR two regression equations are modelled simultaneously (i.e., one for total costs and one for QALY), while correcting for their possible correlation through correlated error terms[16, 34]:

$${Costs}_{i}= \beta_{0c}+ \beta_{1c}{trt}_{i}+ \beta_{42}{uT0}_{i}+ \beta_{3c}{MS}_{i} +\varepsilon_{ci}$$

$${QALYs}_{i}= \beta_{0q}+ \beta_{1q}{trt}_{i}+ \beta_{2q}{uT0}_{i}+ \beta_{3q}{MS}_{i}+\beta_{4q}{HC}_{i}+ \varepsilon_{qi}$$

$\left( \begin{aligned} \varepsilon_{ci} \\ \varepsilon_{qi} \end{aligned} \right)\sim Normal\left( \left( \begin{aligned} 0 \\ 0 \end{aligned} \right), \left( \begin{matrix} \sigma_{c}^{2} & \sigma_{cq} \\ \sigma_{cq} & \sigma_{q}^{2} \end{matrix} \right) \right)$

**Mean Imputation combined with SUR (M-SUR)** – In this strategy, missing cost and utility data were replaced by the mean values from the available cases at each time point (i.e., unconditional mean imputation)[5]. Subsequently, total costs and QALYs were calculated, and SUR analyses were performed as outlined under SUR.

**Multiple Imputation combined with SUR (MI-SUR)** – In this strategy, missing cost and utility data were first imputed using Multivariate Imputation by Chained Equations (MICE) as outlined above. Then, total costs and QALYs were calculated and a SUR was fitted per imputed dataset as outlined under SUR, after which pooled estimates were obtained using Rubin’s rules[32]. The SUR model as fitted as shown by SUR equations above.


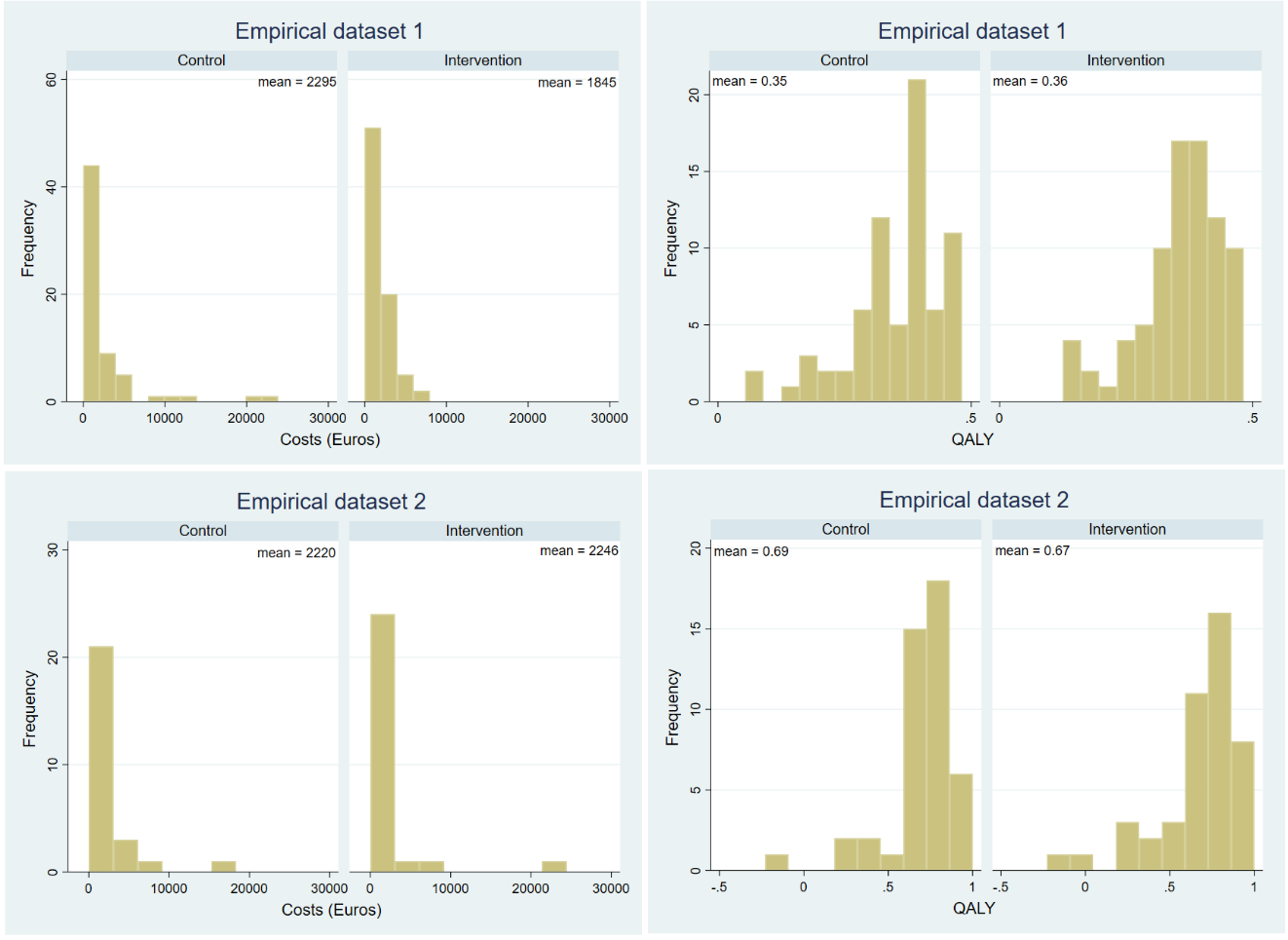


**Supplementary Figure 1.** QALYs and total cost distributions for the control and the intervention groups in empirical dataset 1 and empirical dataset 2.

**Supplementary Table 2. Descriptive statistics of the empirical datasets**

|  | **n** | **uT0,**  **mean (SD)** | **n** | **uT1**  **mean (SD)** | **n** | **uT2**  **mean (SD)** | **n** | **cT1**  **mean (SD)** | **n** | **cT2**  **mean (SD)** | **n** | **cT3**  **mean (SD)** |
| --- | --- | --- | --- | --- | --- | --- | --- | --- | --- | --- | --- | --- |
| **Empirical data 1, = 169** | | | | | | | | | | | | |
| Control |  |  |  |  |  |  |  |  |  |  |  |  |
| Complete | 63 | 0.43 (0.29) | 63 | 0.82 (0.21) | 63 | 0.78 (0.25) | 63 | 685 (1011) | 63 | 790 (2741) | 63 | 819 (1769) |
| Missings | 14 | 0.29 (0.22) | 10 | 0.70 (0.27) | 8 | 0.73 (0.27) | 9 | 1444 (1595) | 8 | 2637 (4128) | 5 | 1215 (1031) |
| Intervention |  |  |  |  |  |  |  |  |  |  |  |  |
| Complete | 78 | 0.41 (0.30) | 78 | 0.81 (0.22) | 78 | 0.78 (0.25) | 78 | 834 (744) | 78 | 495  (742) | 78 | 515 (820) |
| Missings | 14 | 0.35 (0.32) | 6 | 0.83 (0.18) | 4 | 0.92 (0.09) | 5 | 1586 (1975) | 4 | 20 (39) | 3 | 79 (137) |
| **Empirical data 2, n = 143** | | | | | | | | | | | | |
| Control | **n** | **uT0,**  **mean (SD)** | **n** | **uT1**  **mean (SD)** | **n** | **uT2**  **mean (SD)** | **n** | **cT1**  **mean (SD)** | **n** | **cT2**  **mean (SD)** | **n** | **cT3**  **mean (SD)** |
| Complete | 24 | 0.61 (0.29) | 24 | 0.65 (0.32) | 24 | 0.64 (0.22) | 24 | 601 (764) | 24 | 1162 (2590) | 24 | 506 (684) |
| Missings | 45 | 0.60 (0.32) | 32 | 0.75 (0.22) | 21 | 0.68 (0.25) | 45 | 383 (409) | 14 | 1511 (3932) | 5 | 5515 (8749) |
| Intervention |  |  |  |  |  |  |  |  |  |  |  |  |
| Complete | 24 | 0.63 (0.29) | 24 | 0.69 (0.27) | 24 | 0.59 (0.24) | 24 | 591 (1143) | 24 | 381 (373) | 24 | 1441 (4045) |
| Missings | 50 | 0.61 (0.37) | 38 | 0.66 (0.31) | 21 | 0.65 (0.29) | 50 | 418 (365) | 12 | 274 (299) | 8 | 2692 (6281) |

uT0: utility at baseline. uT1: utility at time point 1. uT2: utility at time point 2. cT1: costs at time point 1. cT2: costs at time point 2. cT3: costs at time point 3. Costs were not collected at baseline in both randomized clinical trials.


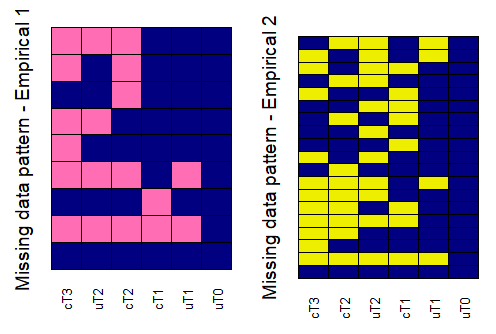


**Supplementary Figure 2.** Missing data pattern in empirical dataset 1 (pink) and empirical dataset 2 (yellow), respectively. Blue squares represent complete data. Coloured squares represent missing data. uT0: utility at baseline. uT1: utility at time point 1. uT2: utility at time point 2. cT1: costs at time point 1. cT2: costs at time point 2. cT3: costs at time point 3.
